# Uncovering the determinants of high-risk age at childbirth in Bangladesh: A machine learning analysis of the BDHS 2022 data

**DOI:** 10.1371/journal.pone.0356362

**Published:** 2026-08-20

**Authors:** A. T. M. Ahsanul Kyum Siam, Tanzila Tanjim Tusra, Md. Abul Hossain

**Affiliations:** Department of Statistics, Mawlana Bhashani Science and Technology University, Tangail, Bangladesh; National Research Centre, EGYPT

## Abstract

Childbirth occurring at high-risk maternal ages (≤18 years or ≥40 years) remains an important public health concern in Bangladesh and is influenced by a range of socio-demographic and behavioral factors. We conducted a cross-sectional secondary analysis of 15,386 ever married women from the BDHS 2022 dataset. After cleaning and removing duplicates and missing values, data were split into training 80 percent and testing 20 percent sets. Three feature selection methods Lasso with 12 features, Chi Square with 13 features, and Boruta with 10 features were applied. Machine learning models including non-ensemble models CART, SVM, KNN, Naive Bayes and ensemble models Random Forest, GBM, AdaBoost, XGB were trained using 10-fold stratified cross validation with SMOTE applied to balance classes. Model performance was evaluated using accuracy, precision, recall, F1 score, Area Under the Receiver Operating Characteristic Curve (AUROC), and Area Under the Precision-Recall Curve (AUPRC).. Among the evaluated machine learning models, CART achieved the best overall performance for the Lasso-selected features (Accuracy = 0.77, F1-score = 0.69, AUROC = 0.74, AUPRC = 0.54), while GBM performed best for the Chi-square-selected features (Accuracy = 0.80, F1-score = 0.71, AUROC = 0.79, AUPRC = 0.64) and Boruta-selected features (Accuracy = 0.75, F1-score = 0.64, AUROC = 0.69). Overall, the GBM model with Chi-square-selected features demonstrated the strongest predictive performance, achieving the highest AUROC (0.79) and F1-score (0.71). Key predictors of high-risk age at childbirth included maternal age, early marriage, current contraceptive use, husband occupation, number of children, husband age and education, respondent age, and regional disparities. Findings were consistent with prior South Asian and LMIC studies while highlighting the added relevance of partner education and media exposure. Machine learning approaches identified the most influential socio demographic and behavioral determinants of high-risk age at childbirth in Bangladesh. The results provide an evidence-based framework to guide policymakers in designing interventions such as delaying age at first birth, expanding female education, and targeting high risk districts to improve maternal and child health outcomes.

## Introduction

The age at first childbirth is a critical demographic indicator that substantially influences a woman’s reproductive lifespan, fertility patterns, and maternal and child health outcomes [1]. Although global adolescent birth rates have declined, teenage pregnancies remain prevalent in many developing countries and exhibit considerable regional variation [2]. High-risk age at childbirth is associated with adverse outcomes, including low birth weight, preterm delivery, and reduced Apgar scores [2,3]. Adolescent mothers also face elevated risks of maternal mortality, infections, and long-term health complications, along with reduced educational attainment and limited socioeconomic opportunities [2,4]. In contrast, delayed first childbirth is generally associated with improved child outcomes, such as better educational performance and fewer behavioral problems, largely due to increased maternal resources and preparedness [5,6]. However, advanced maternal age may also increase the likelihood of obstetric complications, including assisted vaginal deliveries, cesarean sections, and neonatal transfers [7,8].

In Bangladesh, early marriage remains a key social and demographic driver of early childbearing. The Bangladesh Demographic and Health Survey (BDHS) 2017–18 reported a median age at first marriage of 18.6 years among women aged 20–24, reflecting the persistence of early unions [9,10]. This pattern contributes to high-risk age at childbirth, with approximately 55% of ever-married women giving birth before the age of 18 and a mean age at first birth of 18.2 years [10]. Furthermore, disparities persist between urban and rural populations, with rural women more likely to experience earlier first births [2]. These patterns underscore the continued public health and developmental significance of early childbearing in Bangladesh [11].

A substantial body of literature has identified key determinants influencing the timing of first childbirth. Higher educational attainment for both women and their partners is strongly associated with delayed first birth [1,4,5,12,]. In contrast, rural residence, younger partner age, and behavioral factors such as smoking during pregnancy are linked to earlier childbearing [1,4]. Socioeconomic disparities also play a significant role, with evidence indicating a widening gap in maternal age at first birth between high- and low-socioeconomic status groups [5]. Additionally, access to mass media and knowledge of contraceptive methods are important enabling factors that empower women to postpone childbirth [4]. Other contributing factors include marital status, with unmarried adolescent mothers being more prevalent in certain contexts [2], as well as limited access to prenatal care, lower educational attainment, and psychosocial challenges such as anxiety, depression, and inadequate social support [2,13]. Evidence from Ethiopia and sub-Saharan Africa further indicates that the average age at first childbirth remains around 18–19 years, below the optimal range of the late 20s to early 30s [4,5,14]. Collectively, these findings highlight the complex interplay of social, economic, and behavioral determinants shaping early childbearing patterns.

Despite extensive research on the determinants and consequences of high-risk age at childbirth, important gaps remain in the application of advanced analytical approaches to this issue in Bangladesh. No previous study has systematically compared machine learning algorithms to predict early first birth using BDHS 2022 data. Addressing this gap, the present study aims to identify the key determinants of high-risk age at childbirth and evaluate the predictive performance of multiple machine learning models using the most recent nationally representative dataset. By leveraging machine learning techniques, this study provides a more robust and data-driven understanding of high-risk age at childbirth dynamics and offers actionable insights for targeted policy and intervention strategies. The objective of this study is to identify the key determinants to risk at early birth in Bangladesh using the BDHS 2022 dataset. The study intends to provide an evidence based analytical understanding of the socio-demographic and behavioral factors influencing high-risk age at childbirth, supporting targeted interventions to improve maternal and child health outcomes.

## Methods and materials

### Data description

The data for this study were obtained from the Bangladesh Demographic and Health Survey (BDHS) 2022, the most recent in a series of nationally representative surveys conducted every three to four years since 1993. The BDHS surveys employed a two-stage stratified random sample method to make sure that all of Bangladesh's administrative divisions were included. The BDHS surveys ask almost the same questions each time, which lets you see how demographic and health factors, such maternal education, change over time. Previous papers have provided extensive information regarding the BDHS sampling methodology and strategies. The authors did not gather any new data. The Bangladesh Demographic and Health Survey (BDHS) provided the data for the analysis. This survey gathers detailed information about women's lives. We looked at women who had been married at least once and were between the ages of 15 and 49. The study did not include women who had never been married. We looked at sociodemographic and household-level characteristics to see if they were linked to age at first birth.

### Sample size and handling missing data

For this study, we accessed the publicly available BDHS 2022 dataset **(**https://dhsprogram.com/data/available-datasets.cfm**)**, which is nationally representative and covers the entire country.The initial dataset consisted of 30,078 observations of ever-married women. As the primary objective of this study was to develop robust machine learning models using reliable and consistent inputs, only complete-case data were considered for analysis. Therefore, no imputation techniques were applied, as imputation may introduce additional assumptions and potential bias, particularly when the mechanism of missingness is uncertain and when model interpretability is a concern [15].

Accordingly, all records containing missing values (**n = 14,040**) were excluded, resulting in a reduced dataset of **16,038 observations**. Subsequently, **652 duplicate entries** were identified and removed to prevent redundancy and avoid distortion in model training. After these preprocessing steps, the final analytical sample comprised **15,386 complete and unique observations**, which were used for all subsequent analyses.

### Target variable

In this study, high-risk age at childbirth was considered the outcome variable. The variable was coded as **1 (high risk)** for women who gave birth at **≤ 18 years** or **≥ 40 years**, and **0 (low risk/normal)** for women who gave birth between **19 and 39 years** [16,17].

### Addressing class imbalance

**Fig 1:**
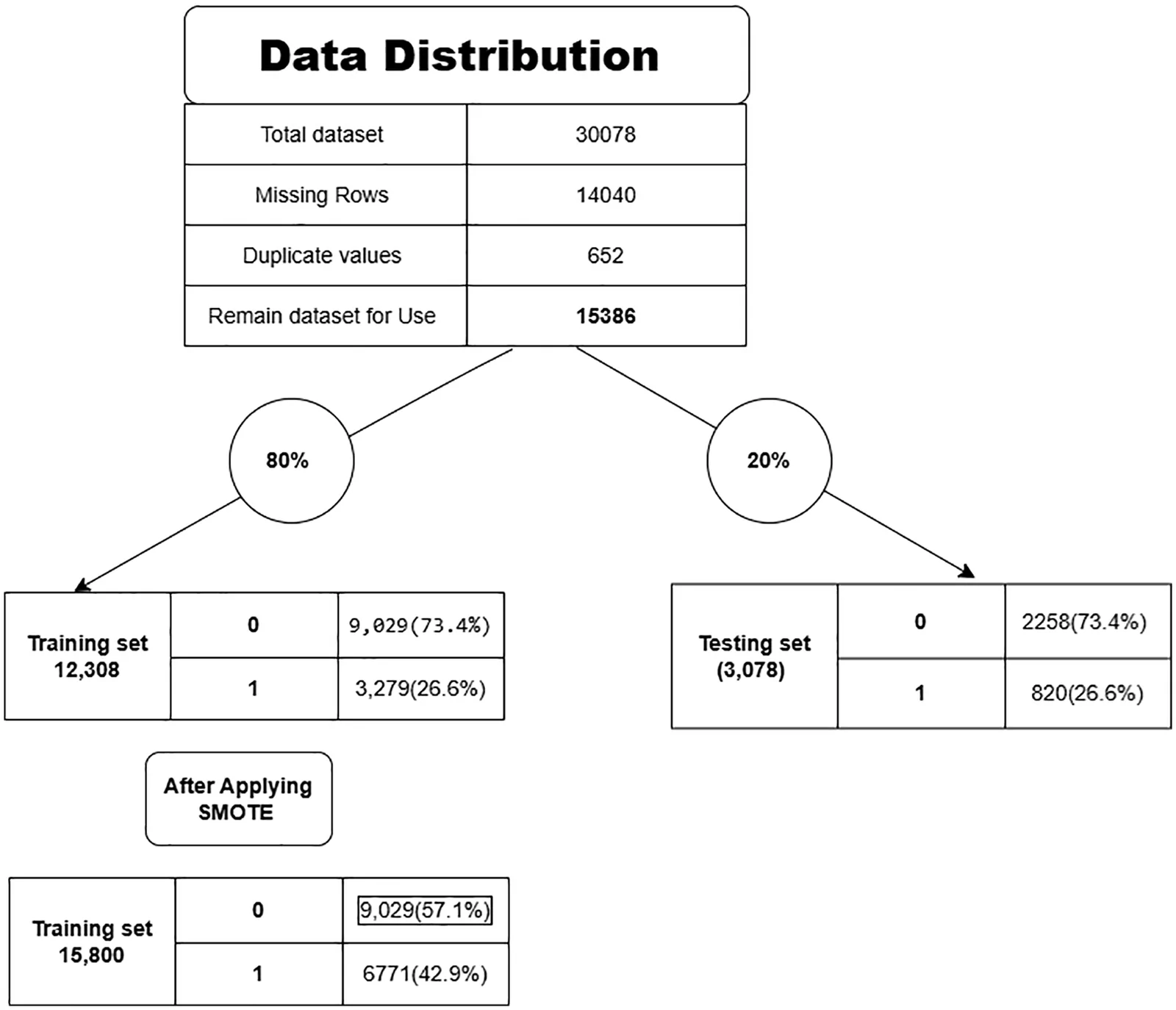
Data Distribution of target variable (high-risk age at childbirth).

It was very critical to fix the class imbalance at the data preprocessing stage. The initial dataset had a huge bias: 9,029(73.4%) in NO (0) and 3,279(26.6%) in Yes (1) details shows in Fig 1. We employed the Synthetic Minority Over-sampling Technique (SMOTE) to remedy this problem in Fig 1. It helped us acquire a more balanced distribution of classes, with a fair 50:50 ratio.

### Independent variablesv

Table 1 presents the 16 variables selected from the Bangladesh Demographic and Health Survey (BDHS) dataset for this study. The variables encompass respondents’ socio-demographic characteristics, household attributes, reproductive factors, fertility preferences, and husband-related characteristics. An additional column is included to display the original BDHS variable codes corresponding to each feature. Variables were classified as categorical or binary, and their coding schemes, value ranges, and missing values are provided to facilitate data preprocessing, analysis, and interpretation.

**Table 1:** Description and coding of Selected Variables from (BDHS) variables used in the analysis.

BDHS Data Code	Features Name	Missing Values	Type	Range
v012	Age of respondent	0	Categorical	0 = 15–18, 1 = 19–25, 2 = 26–30, 3 = 31–35, 4 = 36–39, 5 = 40–45, 6 = 46–49
v025	Place of residence	0	Categorical	0 = Urban, 1 = Rural
v101	Division	0	Categorical	0 = Barisal, 1 = Chittagong, 2 = Dhaka, 3 = Khulna, 4 = Mymensingh, 5 = Rajshahi, 6 = Rangpur, 7 = Sylhet
v106	Respondent Highest educational level	0	Categorical	0 = No education, 1 = Primary, 2 = Secondary, 3 = Higher
v130	Religion	0	Categorical	0 = Islam, 1 = Hinduism, 2 = Christianity, 3 = Buddhism, 4 = Others
v152	Husband age / Age of household head	0	Categorical	0 = 17–30, 1 = 31–50, 2=>50
v155	Literacy	0	Categorical	0 = Cannot read, 1 = Reads part, 2 = Reads full sentence
v190	Wealth index	0	Categorical	0 = Poorest, 1 = Poorer, 2 = Middle, 3 = Richer, 4 = Richest
v212	Risk at pregnancy (birth age)	3145	Binary	0 = Normal, 1 = High risk
v364	Knowledge of contraceptive method	10091	Binary	0 = No, 1 = Yes
v511	Early marriage	10091	Binary	0 = No, 1 = Yes
v613	Ideal number of children	10091	Binary	0=≤2, 1=≥3
v621	Husband desire for child	12109	Categorical	0 = Same, 1 = Husband wants more, 2 = Husband wants fewer, 3 = Don’t know
v623	Exposure	10091	Categorical	0 = Fecund, 1 = Pregnant, 2 = Postpartum amenorrheic, 3 = Infecund
v701	Husband education	11091	Categorical	0 = No education, 1 = Primary, 2 = Secondary, 3 = Higher
v705	Occupation of husband	11091	Categorical	0 = Did not work, 1 = Professional, 2 = Agricultural, 3 = Skilled manual

### Research design

This study employed a cross-sectional design using nationally representative survey data to examine the determinants and predict pregnancy risk among women. Risk of birth at pregnancy was defined based on maternal age at child birth, where women aged ≤18 years or  ≥ 40 years were considered high-risk, and those aged 19–39 years were considered low-risk. The analysis included socio-demographic and health-related factors, and statistical as well as machine learning methods were applied to identify key predictors and estimate the likelihood of high-risk pregnancy.

### Study framework

**Fig 2:**
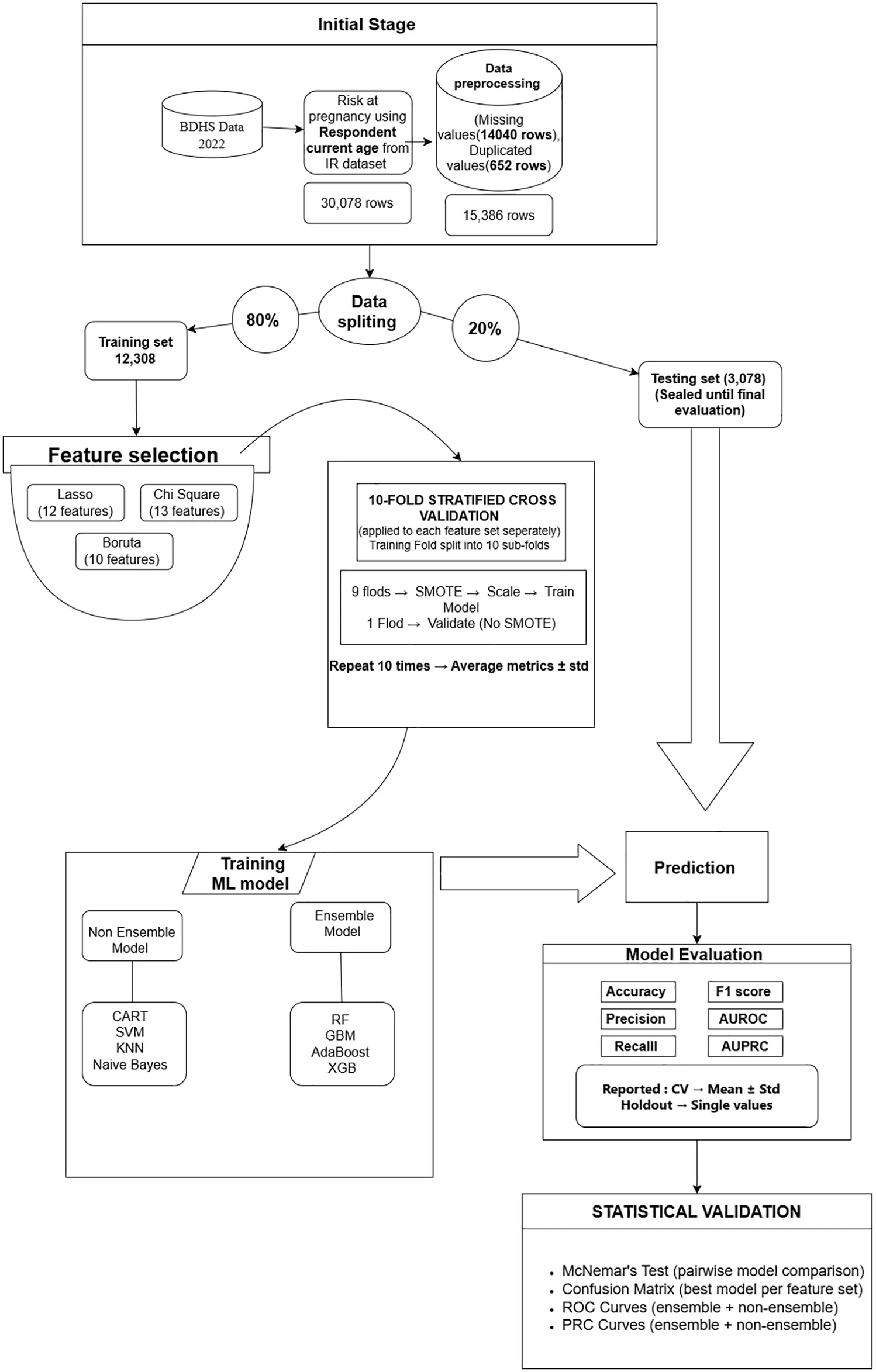
Overview of the study framework, including data preprocessing, feature selection, model development, and evaluation pipeline.

The overall analytical framework of the study is presented in Fig 2. The analysis began with the BDHS 2022 dataset, consisting of 30,078 observations. Pregnancy risk was constructed using the respondent’s current age from the IR dataset. During data preprocessing, 14,040 observations with missing values and 652 duplicate records were removed, resulting in a final dataset of 15,386 observations.

The processed data were then split into training (80%; n = 12,308) and testing (20%; n = 3,078) sets, where the testing set was sealed until the final evaluation stage. Feature selection was conducted on the training data using LASSO (12 features), Chi-square (13 features), and Boruta (10 features) methods.

To ensure model robustness, a 10-fold stratified cross-validation approach was applied. In each iteration, 9 folds were used for training with SMOTE and feature scaling, while 1 fold was used for validation without SMOTE. This process was repeated 10 times, and average performance metrics along with standard deviation were computed.

Subsequently, multiple machine learning models were trained, including non-ensemble methods (CART, SVM, KNN, and Naïve Bayes) and ensemble methods (Random Forest, GBM, AdaBoost, and XGB). Model predictions were evaluated on the testing set using accuracy, precision, recall, F1-score, AUROC, and AUPRC. Finally, statistical validation was performed using McNemar’s test, confusion matrix analysis, ROC curves, and precision–recall curves to compare model performance.

### Feature selection

We also used three different ways to choose features. We used the Least Absolute Shrinkage and Selection Operator (LASSO) [18], Chi square [19], and Boruta feature selection methods [20] to find the most important factors that lead to risk of birth. At the end, we found 12, 13, and 10 significant variables respectively, which are shown in Table 3. This strict method makes our results more reliable and applicable to other situations, giving us useful information on what causes risk of birth.

## Non-ensemble machine learning algorithms

### Classification and regression trees

In 1984, Classification and Regression Trees, Breiman et al. developed the classification and regression trees (CART) algorithm 1984 [21]. Regression and classification issues can now be learned by machines in a novel and significant method. For both classification and regression problems, it produces binary decision trees. The first step is to divide the dataset into smaller subsets based on the attributes’ values. This ensures that the goal variable is as comparable across all groups as feasible. Mean squared error (MSE) reduction is used for regression, and CART uses metrics like entropy or Gini impurity to find the best splits for classification. Through repeated division, a tree is created, with the core nodes displaying feature assessments and the leaf nodes displaying class labels or continuous values.

### Support vector machine

A support vector machine (SVM) finds a hyperplane in a multidimensional space that acts as a decision boundary [22]. In order to facilitate separation, this hyperplane was selected to maximize the distance between data points from various classes. The maximum margin approach strengthens the model's resistance to overfitting and noise. Support vector machines, which were originally created for linear datasets, utilize kernel functions such as Gaussians to manage nonlinear data by transforming it into higher dimensions, which facilitates linear separation. The adaptability of these methods makes them suitable for a range of applications, including picture and text classification [23]

### Naive Bayes

Naive Bayes is a fundamental and effective machine learning technique predicated on supervised categorization. This method is a component of Bayesian decision theory. Naive Bayes uses Bayes’ theorem to compute the likelihood of a class given the features [24]. The formula is as follows:


$$\begin{array}{c} P(X)=\frac{P(C).P(C)}{P(X)} \end{array}$$

P(C|X) denotes the posterior probability of class C given attributes X, P(X|C) represents the likelihood, P(C) signifies the prior probability, and P(X) indicates the evidence.

We use Bernoulli naive bayes with an alpha value of 10 in this study because the target variable has two categories. We obtain the best results by changing the parameters to an alpha score of 10. Naive Bayes algorithms are of different types: Gaussian Naive Bayes and multinomial Naive Bayes are two prevalent methods for categorizing text into groups. The Naive Bayes algorithm can be used for: Naive Bayes is a simple but useful way to sort things. It does a great job of handling large amounts of data. It works well sorting through spam, determining how people feel about text, and testing recommendation systems.

### K-nearest neighbors (KNN)

The k-nearest neighbors (k-NN) algorithm is a straightforward and efficient supervised learning technique applicable to both classification and regression tasks [25]. This method is nonparametric and instance-based, making predictions by locating the k nearest data points in the training set. In classification, it designates the most prevalent class among the neighbors, whereas in regression, it forecasts the mean of their values. The algorithm generally employs distance metrics such as Euclidean or Manhattan distances to assess similarity. This approach necessitates no training phase, categorizing it as a lazy learner; however, this characteristic can lead to high computational costs during the prediction phase. The k-NN achieves optimal performance when features are normalized, because of its sensitivity to scale. This approach finds extensive use in fields such as medical diagnosis, pattern recognition, and recommendation systems; however, it may encounter challenges when dealing with noisy or high-dimensional data [26]

The Manhattan distance measurement equation is given below:


$$\begin{array}{c} M_{e}=\sum_{i=\text{1}}^{n}\left|a_{i}-b_{i}\right| \end{array}$$

Here,

a_i_= points of group a

b_i_ = points of group b

## Ensemble machine learning algorithms

### Random forest

Introduced by Breiman in 2001 [27] this method has become highly favored in the field of machine learning because of its ability to manage high-dimensional data, understand complex relationships among variables, and deliver reliable predictions consistently. Owing to its simplicity, effectiveness, and adaptability, the Random Forest continues to be a favored option for classification and regression tasks across various domains, providing a powerful tool for predictive modeling and analysis [28].

### Extreme Gradient Boosting (XGB)

XGB, created by Tianqi Chen and Carlos Guestrin, is a robust ensemble learning algorithm recognized for its efficiency, precision, and scalability [29,30]. To create a strong prediction model, gradient boosting combines many weak learners, usually decision trees [31]. XGB excels at managing big datasets because of its efficient design, which permits distributed and parallel processing. In order to combat overfitting and broaden the model's scope, it also incorporates regularization techniques. For both classification and regression problems, XGB is frequently utilized. Its effectiveness and adaptability to a wide range of scenarios make it a popular option for machine learning contests and industrial applications.

### Adaptive Boosting (AdaBoost)

AdaBoost (Adaptive Boosting) is an ensemble learning technique created by Freund and Schapire in 1996 to improve classification accuracy by integrating numerous weak learners, usually decision stumps [32]. It operates iteratively, initially allocating uniform weights to all training samples. Following each iteration, increased weights are assigned to misclassified instances, prompting the following models to concentrate on more challenging examples. This procedure persists until a robust classifier is constructed from the series of weak classifiers. AdaBoost efficiently diminishes bias and variance, rendering it resilient to overfitting. It is very efficacious in binary classification tasks and has strong performance on imbalanced or noisy datasets. Its simplicity and high accuracy have made it a popular choice for text classification and medical applications.

### Model evaluation metrics

By comparing actual events to projected outcomes, the confusion matrix gives a full picture of how well a categorization works [33]. We selected the following key metrics to provide a more comprehensive evaluation.

### Accuracy

The percentage of correctly identified results over the total number of predictions is known as accuracy.


$$\begin{array}{c} Accuracy=\left(\frac{Tp+Tn}{Tp+Tn+Fp+Fn}\right) \end{array}$$

### Recall

The percentage of true positives the classifier successfully receives from the whole amount of test data is known as recall/sensitivity.


$$\begin{array}{c} Recall=\left(\frac{Tp}{Tp+Fn}\right) \end{array}$$

### Precision

Precision is the fraction of correctly positive prediction overall positive predictions achieved by the classifier.


$$\begin{array}{c} Precision=\left(\frac{Tp}{Tp+Fp}\right) \end{array}$$

### F1 score

The F1 Score is the metric used to check for precision and recall alignment.


$$\begin{array}{c} F\text{1}=\text{2}\times \frac{Precision\times Recall}{Precision+Recall} \end{array}$$

### Receiver operating characteristic (ROC) curve and area under the curve (AUC)

The ROC curve shows the balance between true positive and false positive rates at different levels of classification. The Area Under the Curve (AUC) is a measure of how well a model works. Higher AUC values mean that the model can classify things better. This number is very important for judging machine learning systems that make predictions about fertility.

### Precision recall curve

The precision–recall (PR) curve is a widely used evaluation tool for classification models, particularly in imbalanced datasets where accuracy can be misleading. It illustrates the trade-off between precision (positive predictive value) and recall (sensitivity) across different decision thresholds. A model that maintains high precision while achieving high recall is generally preferred, indicating robust performance in identifying true positive cases with minimal false positives. The area under the precision–recall curve (AUPRC) provides a single summary metric, with higher values reflecting better model discrimination. This approach has been strongly recommended in high-impact journals for evaluating predictive models in imbalanced settings [34, 35]

### McNemar's test

McNemar’s test is a non-parametric statistical method used to compare the performance of two classification models on the same dataset, particularly when the outcomes are paired and categorical. It focuses on the discordant pairs—instances where the two models make different predictions—to determine whether there is a statistically significant difference in their performance. The test is based on a 2 × 2 contingency table and evaluates the null hypothesis that both models have the same error rate. A chi-square statistic is computed using the counts of disagreements, and a p-value is obtained to assess significance. McNemar’s test is widely recommended in machine learning research for comparing classifiers on paired data [36].

### Ethics approval

This research is based on secondary data from the Bangladesh Demographic and Health Survey (BDHS) 2022, made available through The DHS Program (The DHS Program - Quality information to plan, monitor and improve population, health, and nutrition programs). The original data collection process received ethical clearance from the Institutional Review Board (IRB) of ICF International in Calverton, Maryland, USA, as well as from the appropriate ethics committees in Bangladesh. As this study involves the analysis of anonymized, publicly accessible data, no additional ethical approval was required. The datasets can be accessed by registering online via The DHS Program website. (The DHS Program - Access Instructions)

## Results

### Experimental settings

All experiments were conducted using Python (version 3.11) on the Google Colaboratory (Colab) platform, utilizing an NVIDIA T4 GPU, Intel Xeon 2.20 GHz CPU, and 12 GB RAM. Data preprocessing and manipulation were performed using Pandas (version 2.2.2) and NumPy (version 2.0.2), while visualization was conducted using Matplotlib (version 3.5.2) and Seaborn (version 0.12.2). Machine learning models, including Logistic Regression (LR), Classification and Regression Trees (CART), Random Forest (RF), Gradient Boosting Machine (GBM), Extreme Gradient Boosting (XGB), and K-Nearest Neighbors (KNN), were implemented using Scikit-learn (version 1.6.1) and XGB (version 2.0.2), with additional deep learning support from TensorFlow (version 2.12).

Model performance was evaluated using 10-fold cross-validation to ensure robust generalization. To address class imbalance, the Synthetic Minority Oversampling Technique (SMOTE) was applied using the imbalanced-learn package (version 0.14.1). Importantly, SMOTE was applied only to the training data within each cross-validation fold, thereby preventing data leakage and preserving the integrity of the test data.

### Descriptive analysis

Table 2 presents the descriptive analysis, highlighting several important gradients associated with the outcome. Educational attainment showed a strong inverse relationship, with prevalence highest among women with no education (57.38%) and substantially lower among those with higher education (16.59%). Socioeconomic status also demonstrated a clear pattern, as prevalence increased from 24.31% in the poorest group to 29.12% among the richest. Differences by place of residence were minimal, with similar proportions observed in urban (26.69%) and rural (26.62%) areas, indicating no meaningful disparity. Reproductive factors were particularly influential, as women with three or more children had higher prevalence (34.02%) compared to those with two or fewer (23.99%). Additionally, contraceptive use was significantly associated with the outcome, with higher prevalence among traditional method users (41.07%) compared to modern method users (33.80%) and non-users (19.29%). Overall, these findings suggest that education, wealth, and reproductive behaviors are the most prominent factors linked to the outcome.

**Table 2:** Descriptive Statistics: Frequency, Percentage and Chi-square Test Results with p-values.

Variable	Category	Total n(%)	No n(%)	Yes n(%)	X2 value(df)	P value
Types of places of residence	Urban	5306 (34.49%)	3890 (73.31%)	1416 (26.69%)	0.005(1)	0.941
	Rural	10080 (65.51%)	7397 (73.38%)	2683 (26.62%)	0.005(1)	0.941
Division	Barisal	1695 (11.02%)	1246 (73.51%)	449 (26.49%)	37.091(7)	0.000***
	Chittagong	2352 (15.29%)	1800 (76.53%)	552 (23.47%)	37.091(7)	0.000***
	Dhaka	2282 (14.83%)	1699 (74.45%)	583 (25.55%)	37.091(7)	0.000***
	Khulna	1979 (12.86%)	1389 (70.19%)	590 (29.81%)	37.091(7)	0.000***
	Mymensingh	1694 (11.01%)	1253 (73.97%)	441 (26.03%)	37.091(7)	0.000***
	Rajshahi	1903 (12.37%)	1336 (70.20%)	567 (29.80%)	37.091(7)	0.000***
	Rangpur	1855 (12.06%)	1343 (72.40%)	512 (27.60%)	37.091(7)	0.000***
	Sylhet	1626 (10.57%)	1221 (75.09%)	405 (24.91%)	37.091(7)	0.000***
Highest educational level	No education	2025 (13.16%)	863 (42.62%)	1162 (57.38%)	1347.313(3)	0.000***
	Primary	4253 (27.64%)	2968 (69.79%)	1285 (30.21%)	1347.313(3)	0.000***
	Secondary	7004 (45.52%)	5701 (81.40%)	1303 (18.60%)	1347.313(3)	0.000***
	Higher	2104 (13.67%)	1755 (83.41%)	349 (16.59%)	1347.313(3)	0.000***
Religion	Islam	13727 (89.22%)	10081 (73.44%)	3646 (26.56%)	3.667(4)	0.453
	Hinduism	1462 (9.50%)	1073 (73.39%)	389 (26.61%)	3.667(4)	0.453
	Christianity	161 (1.05%)	109 (67.70%)	52 (32.30%)	3.667(4)	0.453
	Buddhism	32 (0.21%)	21 (65.62%)	11 (34.38%)	3.667(4)	0.453
	Others	4 (0.03%)	3 (75.00%)	1 (25.00%)	3.667(4)	0.453
Literacy	Can not read at all	3297 (21.38%)	1682 (50.96%)	1615 (49.04%)	1183.832(4)	0.000***
	Able to read only parts of sentence	2548 (16.56%)	1820 (71.43%)	728 (28.57%)	1183.832(4)	0.000***
	Able to read Whole sentence	9541 (62.01%)	7785 (81.60%)	1756 (18.40%)	1183.832(4)	0.000***
Wealth index combined	Poorest	2797 (18.18%)	2117 (75.69%)	680 (24.31%)	18.420(4)	0.001**
	Poorer	3054 (19.85%)	2244 (73.48%)	810 (26.52%)	18.420(4)	0.001**
	Middle	3103 (20.17%)	2274 (73.28%)	829 (26.72%)	18.420(4)	0.001**
	Richer	3170 (20.60%)	2340 (73.82%)	830 (26.18%)	18.420(4)	0.001**
	Richest	3262 (21.20%)	2312 (70.88%)	950 (29.12%)	18.420(4)	0.001**
Husband desire for children	Both want same	13409 (87.15%)	9853 (73.48%)	3556 (26.52%)	16.014(3)	0.001**
	Husband wants more	1061 (6.90%)	762 (71.82%)	299 (28.18%)	16.014(3)	0.001**
	Husband wants fewer	552 (3.59%)	430 (77.90%)	122 (22.10%)	16.014(3)	0.001**
	Don’t know	364 (2.37%)	242 (66.48%)	122 (33.52%)	16.014(3)	0.001**
Exposure	Fecund	11167 (72.58%)	8725 (78.13%)	2442 (21.87%)	1783.119(3)	0.000***
	Pregnant	696 (4.52%)	676 (97.13%)	20 (2.87%)	1783.119(3)	0.000***
	Postpartum amenorrheic	862 (5.60%)	769 (89.21%)	93 (10.79%)	1783.119(3)	0.000***
	Infecund, menopausal	2661 (17.29%)	1117 (41.98%)	1544 (58.02%)	1783.119(3)	0.000***
Husband education level	No education	3412 (22.18%)	2037 (59.70%)	1375 (40.30%)	424.862(4)	0.000***
	Primary	4490 (29.18%)	3425 (76.28%)	1065 (23.72%)	424.862(4)	0.000***
	Secondary	4855 (31.55%)	3810 (78.48%)	1045 (21.52%)	424.862(4)	0.000***
	Higher	2629 (16.91%)	2015 (76.63%)	614 (23.37%)	424.862(4)	0.000***
Respondent currently working	Yes	10515 (68.34%)	7772 (73.91%)	2743 (26.09%)	5.137(1)	0.023*
	No	4871 (31.66%)	3515 (72.16%)	1356 (27.84%)	5.137(1)	0.023*
Occupation of husband	Did not work	616 (4.00%)	294 (47.73%)	322 (52.27%)	295.928(3)	0.000***
	Professional	6516 (42.35%)	4783 (73.40%)	1733 (26.60%)	295.928(3)	0.000***
	Agricultural	1694 (11.01%)	1135 (67.00%)	559 (33.00%)	295.928(3)	0.000***
	Skilled Manual	6560 (42.64%)	5075 (77.36%)	1485 (22.64%)	295.928(3)	0.000***
Respondent current age	15-18	409 (2.66%)	0 (0.00%)	409 (100.00%)	15386.000(6)	0.000***
	19-25	3379 (21.96%)	3379 (100.00%)	0 (0.00%)	15386.000(6)	0.000***
	26-30	3110 (20.21%)	3110 (100.00%)	0 (0.00%)	15386.000(6)	0.000***
	31-35	2787 (18.11%)	2787 (100.00%)	0 (0.00%)	15386.000(6)	0.000***
	36-40	2011 (13.07%)	2011 (100.00%)	0 (0.00%)	15386.000(6)	0.000***
	41-45	2485 (16.15%)	0 (0.00%)	2485 (100.00%)	15386.000(6)	0.000***
	46-50	1205 (7.83%)	0 (0.00%)	1205 (100.00%)	15386.000(6)	0.000***
Husband age	17-30	1957 (12.72%)	1742 (89.01%)	215 (10.99%)	1210.259(2)	0.000***
	31-50	8566 (55.67%)	6839 (79.84%)	1727 (20.16%)	1210.259(2)	0.000***
	more than 50	4863 (31.61%)	2706 (55.64%)	2157 (44.36%)	1210.259(2)	0.000***
Number of children	2 or less	11324 (73.60%)	8607 (76.01%)	2717 (23.99%)	153.358(1)	0.000***
	3 or more	4062 (26.40%)	2680 (65.98%)	1382 (34.02%)	153.358(1)	0.000***
Current contraceptive method	NoMethod	8443 (54.87%)	6814 (80.71%)	1629 (19.29%)	551.490(2)	0.000***
	Traditional Method	1697 (11.03%)	1000 (58.93%)	697 (41.07%)	551.490(2)	0.000***
	Modern method	5246 (34.10%)	3473 (66.20%)	1773 (33.80%)	551.490(2)	0.000***

### Feature selection

**Fig 3:**
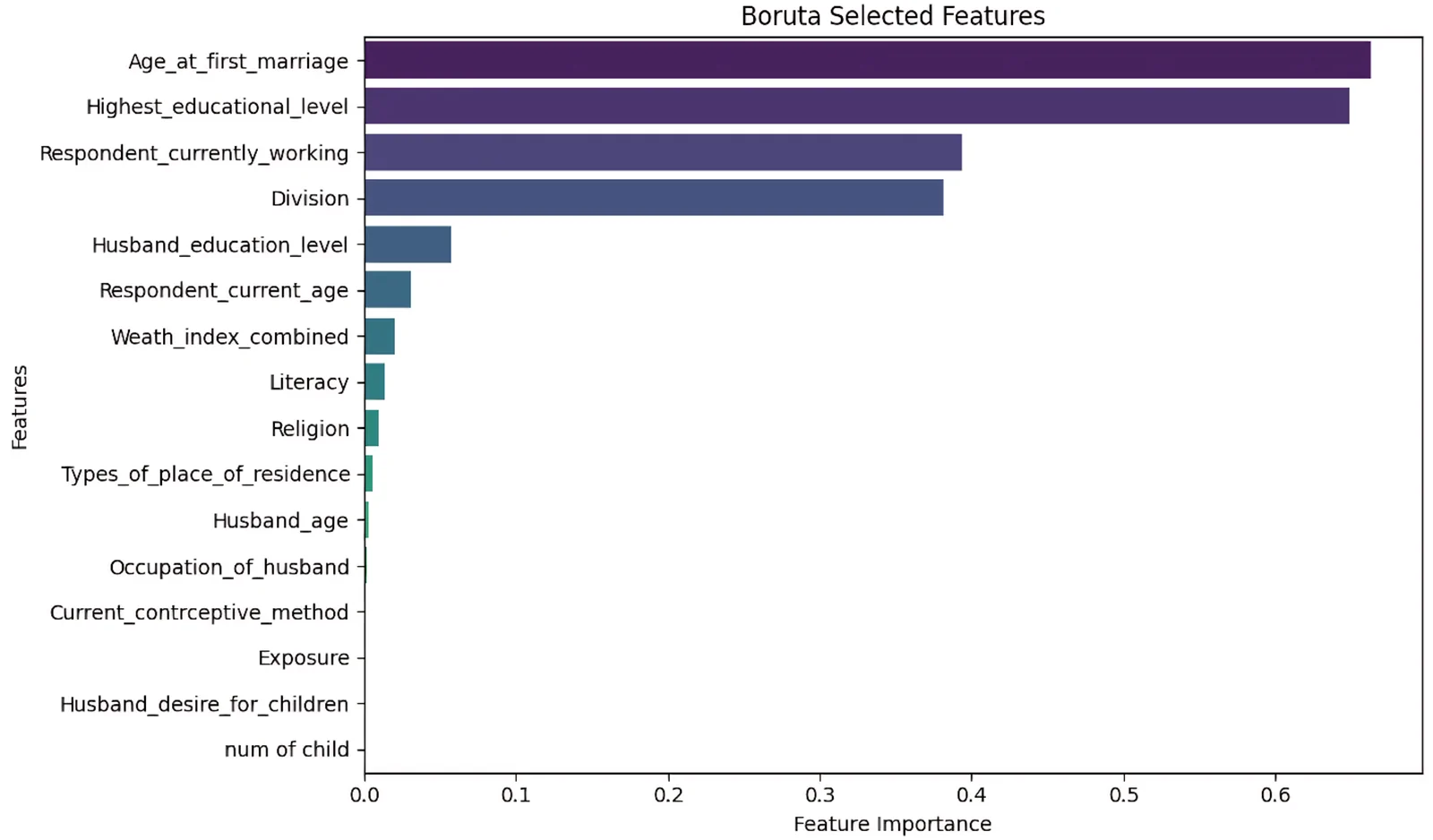
Feature importance of Boruta feature selection technique.

**Fig 4:**
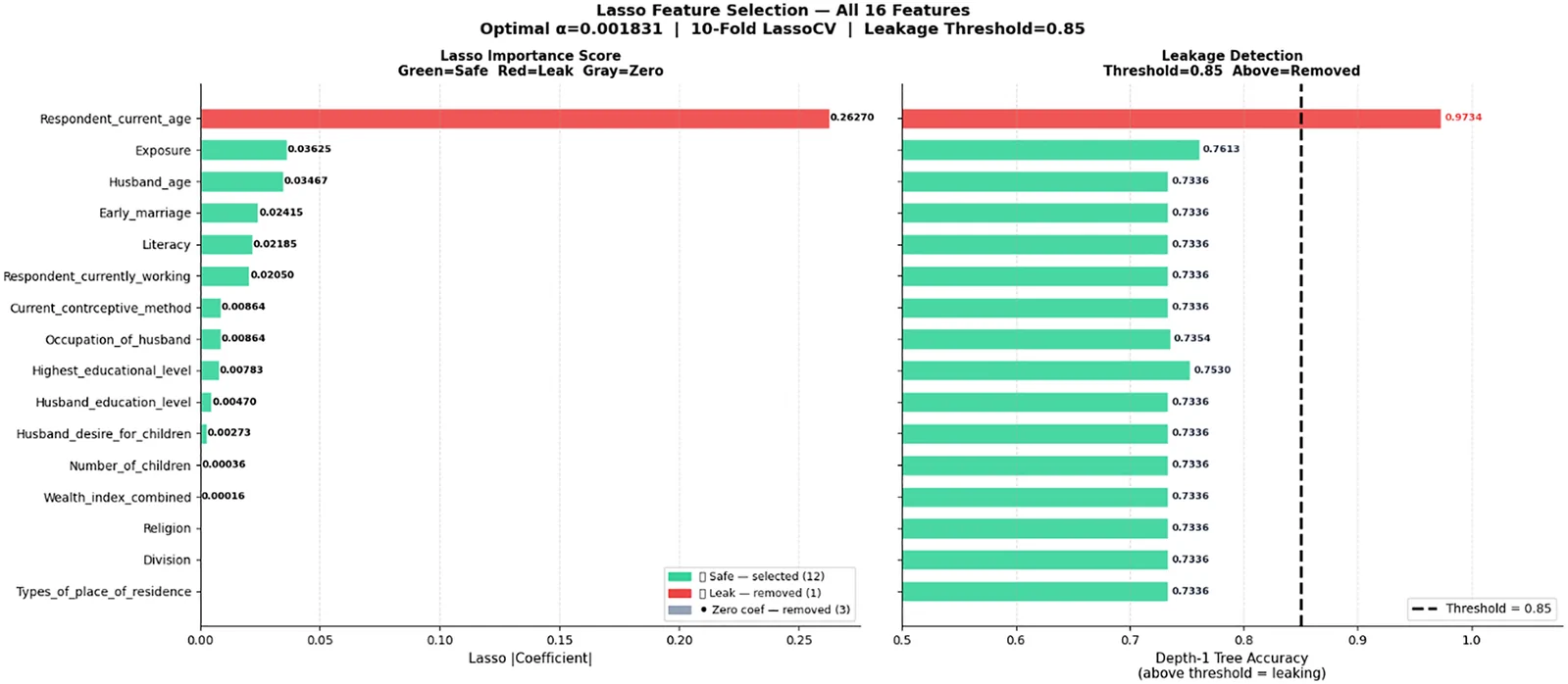
Feature importance of Lasso feature selection technique.

Three different feature selection techniques were applied in the training dataset such as LASSSO, Chi-square and BORUTA. Like a conventional approach we don’t take the common features from those three feature selection techniques, on comparison we create three group of features obtained from those three feature selection techniques and perform machine learning techniques on each feature groups and compare them and try to find out which machine learning models works better with which set of features. Fig 3 illustrates the feature importance rankings derived from the Boruta algorithm, highlighting the significant variables associated with risk of birth at pregnancy prediction. Beside this Fig 4 depicts feature importance for Lasso feature selection technique and alongside this Table 2 shows the associated p-values, chi-square score with degree of freedom for each feature. Based on 95% confidence interval (CI) whose P value less than 0.05 we selected them as chi square selected features...

Using the Lasso method, features with coefficients equal to or near zero were considered insignificant and removed. Only features with non-zero coefficients were retained and are illustrated in Fig 4. In the Boruta feature selection process, variables that failed to exceed the defined importance threshold were excluded. The feature importance rankings are illustrated in Fig 3. Only features classified as *confirmed* were included in the final model, whereas *tentative* features were disregarded. The selected features showed in Table 3, used for comparing ensemble and non-ensemble machine learning models.

**Table 3:** Selected three feature groups from three features selection techniques.

Feature Selection Techniques	Selected Features
F1- Lasso – 12	Exposure, Husband age, Early marriage, Literacy, Respondent currently working, Current contraceptive method, Occupation of husband, Highest educational level, Husband education level, Husband desire for children, Number of children, Wealth index combined
F2- Chi Square – 13	Division, Highest educational level, Literacy, Wealth index combined, Husband desire for children, Exposure, Husband education level, Respondent currently working, Occupation of husband, Respondent current age, Husband age, Number of children, Current contraceptive method
F3- Boruta- 10	Husband’s education level, Division, Respondent Current Age, Wealth index combined, Literacy, Religion, Place of residence, Respondent is currently working, Husband age, Occupation of husband

### Model’s performance comparison

**Fig 5:**
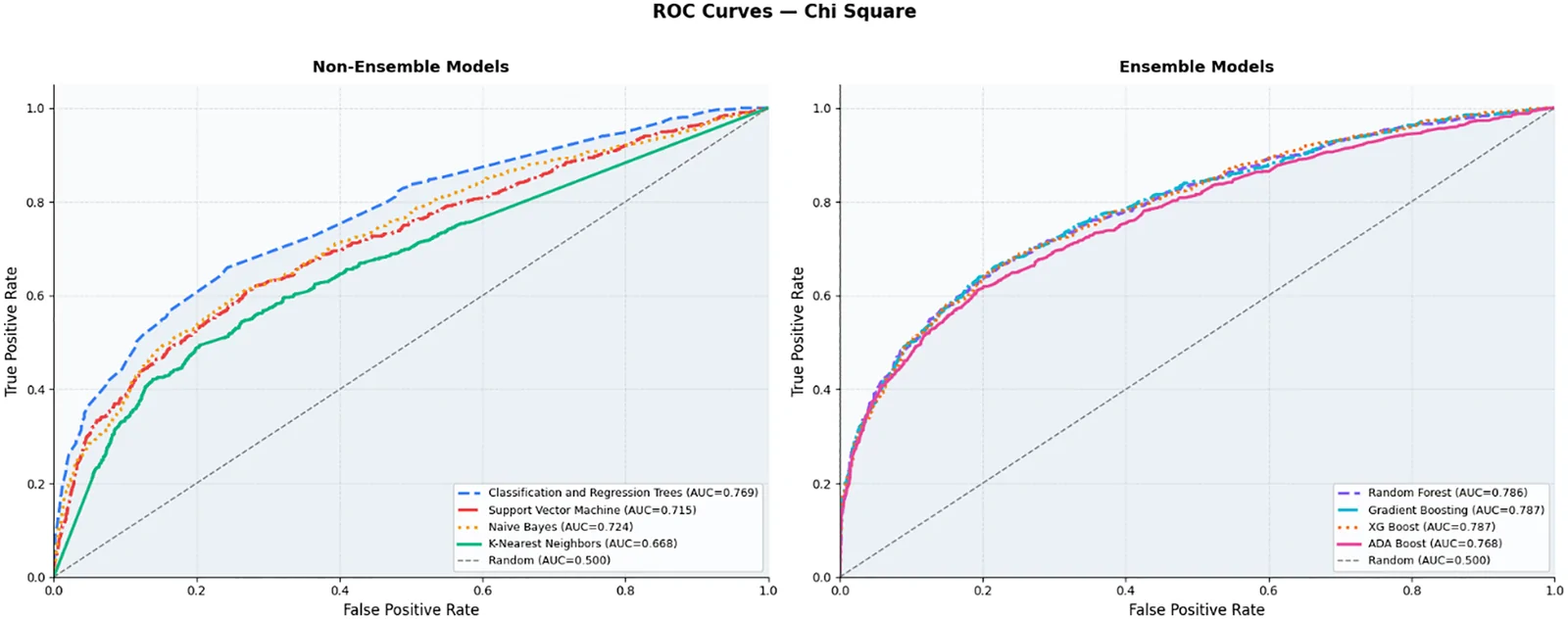
ROC curve of ensemble and non-ensemble machine learning models using Chi square features.

The cross-validation results in Table 4 show that models using Chi-square (F2) selected features consistently outperformed those based on Lasso (F1) and Boruta (F3). As shown in Fig 5, Chi-square-based models exhibited better discriminative performance. The Random Forest model achieved the highest performance (accuracy: 0.7886 ± 0.0107; F1-score: 0.7225 ± 0.0141; AUC: 0.7916 ± 0.0101), followed by Gradient Boosting and XGB with comparable AUC values.

**Table 4:** Cross-validation performance (mean ± SD) of models using Lasso, Chi-square, and Boruta features.

	F1 - Lasso Selected Feature					
	Model	Accuracy (Mean ± SD)	Precision (Mean ± SD)	Recall (Mean ± SD)	F1 Score (Mean ± SD)	AUC Score (Mean ± SD)
**Non ensemble Model**	CART	0.7626 ± 0.0086	0.6938 ± 0.0117	0.6821 ± 0.0134	0.6870 ± 0.0125	0.7418 ± 0.0153
	SVM	0.7623 ± 0.0105	0.6938 ± 0.0141	0.6848 ± 0.0148	0.6888 ± 0.0144	0.7026 ± 0.0128
	NB	0.6696 ± 0.0129	0.6179 ± 0.0140	0.6411 ± 0.0170	0.6202 ± 0.0149	0.6746 ± 0.0225
	KNN	0.7537 ± 0.0165	0.6783 ± 0.0284	0.6314 ± 0.0196	0.6426 ± 0.0222	0.6980 ± 0.0125
**Ensemble Model**	RF	0.7643 ± 0.0084	0.6962 ± 0.0117	0.6858 ± 0.0141	0.6903 ± 0.0130	0.7458 ± 0.0135
	GBM	0.7636 ± 0.0087	0.6954 ± 0.0118	0.6857 ± 0.0138	0.6899 ± 0.0129	0.7453 ± 0.0144
	XGB	0.7627 ± 0.0093	0.6944 ± 0.0127	0.6855 ± 0.0151	0.6893 ± 0.0140	0.7460 ± 0.0142
	AdaBoost	0.7544 ± 0.0125	0.6845 ± 0.0161	0.6798 ± 0.0170	0.6818 ± 0.0163	0.7376 ± 0.0167
	**F2 - CHI Selected Feature**					
	**Model**	**Accuracy (Mean ± SD)**	**Precision (Mean ± SD)**	**Recall (Mean ± SD)**	**F1 Score (Mean ± SD)**	**AUC Score (Mean ± SD)**
**Non ensemble Model**	CART	0.7797 ± 0.0091	0.7177 ± 0.0118	0.7102 ± 0.0120	0.7135 ± 0.0113	0.7720 ± 0.0113
	SVM	0.7537 ± 0.0133	0.6846 ± 0.0172	0.6836 ± 0.0172	0.6841 ± 0.0172	0.7329 ± 0.0147
	NB	0.7248 ± 0.0134	0.6565 ± 0.0156	0.6681 ± 0.0168	0.6611 ± 0.0162	0.7211 ± 0.0200
	KNN	0.7145 ± 0.0119	0.6449 ± 0.0129	0.6561 ± 0.0131	0.6492 ± 0.0130	0.6847 ± 0.0175
**Ensemble Model**	RF	0.7886 ± 0.0107	0.7295 ± 0.0141	0.7174 ± 0.0149	0.7225 ± 0.0141	0.7916 ± 0.0101
	GBM	0.7812 ± 0.0077	0.7197 ± 0.0098	0.7154 ± 0.0124	0.7173 ± 0.0110	0.7899 ± 0.0082
	XGB	0.7823 ± 0.0099	0.7213 ± 0.0124	0.7153 ± 0.0127	0.7179 ± 0.0123	0.7844 ± 0.0091
	AdaBoost	0.7699 ± 0.0078	0.7056 ± 0.0096	0.7041 ± 0.0115	0.7046 ± 0.0102	0.7694 ± 0.0111
	**F3 - Boruta Selected Feature**					
	**Model**	**Accuracy (Mean ± SD)**	**Precision (Mean ± SD)**	**Recall (Mean ± SD)**	**F1 Score (Mean ± SD)**	**AUC Score (Mean ± SD)**
**Non ensemble Model**	CART	0.7155 ± 0.0128	0.6337 ± 0.0167	0.6302 ± 0.0168	0.6316 ± 0.0162	0.6889 ± 0.0197
	SVM	0.7162 ± 0.0125	0.6353 ± 0.0156	0.6329 ± 0.0148	0.6339 ± 0.0150	0.6731 ± 0.0166
	NB	0.6571 ± 0.0195	0.6073 ± 0.0148	0.6287 ± 0.0154	0.6078 ± 0.0171	0.6474 ± 0.0250
	KNN	0.7027 ± 0.0100	0.6039 ± 0.0162	0.5909 ± 0.0157	0.5950 ± 0.0164	0.6245 ± 0.0137
**Ensemble Model**	RF	0.7283 ± 0.0109	0.6464 ± 0.0146	0.6366 ± 0.0136	0.6407 ± 0.0140	0.7024 ± 0.0197
	GBM	0.7201 ± 0.0111	0.6408 ± 0.0136	0.6389 ± 0.0127	0.6398 ± 0.0131	0.6970 ± 0.0155
	XGB	0.7162 ± 0.0115	0.6357 ± 0.0139	0.6339 ± 0.0130	0.6347 ± 0.0133	0.6901 ± 0.0169
	AdaBoost	0.7396 ± 0.0134	0.6579 ± 0.0194	0.6353 ± 0.0151	0.6430 ± 0.0163	0.7006 ± 0.0198

**Fig 6:**
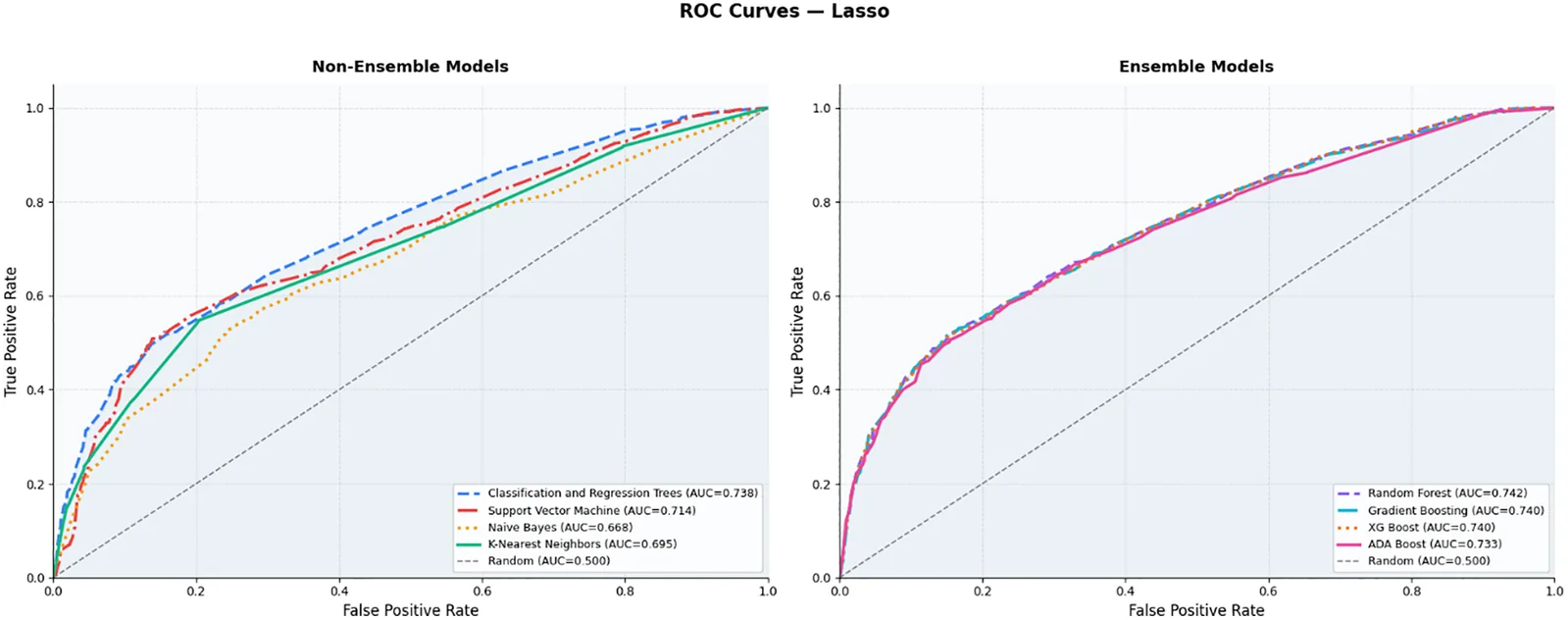
ROC curve of ensemble and non-ensemble machine learning models using Lasso features.

**Fig 7:**
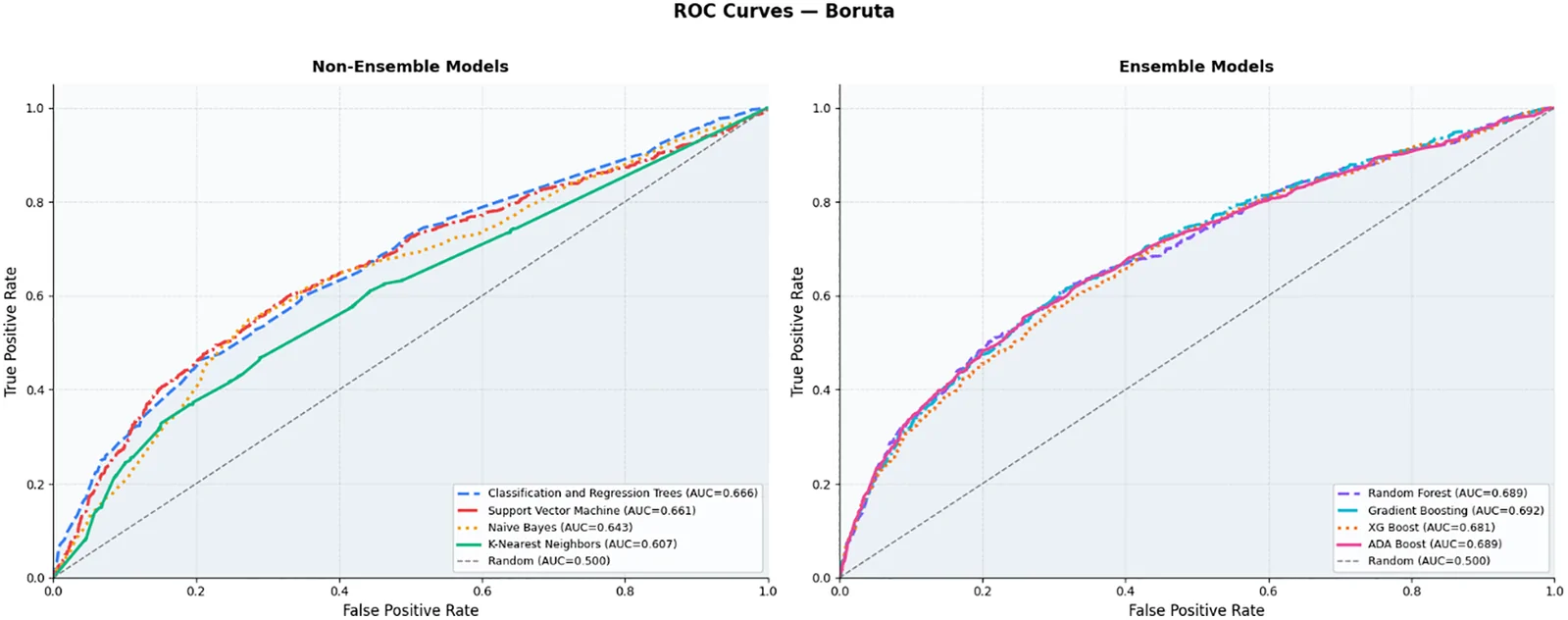
ROC curve of ensemble and non-ensemble machine learning models using Boruta features.

In contrast, models based on Lasso and Boruta features demonstrated lower performance, as reflected in Figs 6 and Fig 7. For Boruta (F3), the best-performing model (AdaBoost) achieved an accuracy of 0.7396 ± 0.0134 and AUC of 0.7006 ± 0.0198.

The test set results in Table 5 are consistent with the cross-validation findings. As illustrated in Fig 5, models using Chi-square features showed better generalization, with Gradient Boosting and XGB achieving the highest accuracy (0.80) and AUC (0.79). Random Forest also performed comparably (accuracy: 0.79; AUPRC: 0.64). In comparison, Lasso (Fig 6) and Boruta (Fig 7) based models showed lower performance, with maximum AUC values of 0.74 and 0.69, respectively.

**Table 5:** Test set performance of models using Lasso, Chi-square, and Boruta features.

	F1 - Lasso Selected Feature						
	**Model**	**Accuracy**	**Precision**	**Recall**	**F1 Score**	**AUC Score**	**AUPRC**
**Non ensemble Model**	CART	0.77	0.70	0.68	0.69	0.74	0.54
	SVM	0.76	0.69	0.68	0.69	0.71	0.49
	NB	0.67	0.62	0.64	0.62	0.67	0.45
	KNN	0.75	0.68	0.63	0.64	0.69	0.47
**Ensemble Model**	RF	0.76	0.69	0.68	0.68	0.74	0.55
	GBM	0.76	0.69	0.68	0.68	0.74	0.55
	AdaBoost	0.75	0.68	0.67	0.68	0.73	0.54
	XGB	0.76	0.69	0.68	0.68	0.74	0.55
	**F2 - CHI Selected Feature**						
	**Model**	**Accuracy**	**Precision**	**Recall**	**F1 Score**	**AUC Score**	**AUPRC**
**Non ensemble Model**	CART	0.78	0.72	0.69	0.70	0.77	0.60
	SVM	0.75	0.67	0.66	0.66	0.71	0.53
	NB	0.73	0.66	0.67	0.66	0.72	0.54
	KNN	0.71	0.64	0.64	0.64	0.67	0.44
**Ensemble Model**	RF	0.79	0.74	0.70	0.71	0.79	0.64
	GBM	0.80	0.75	0.68	0.70	0.79	0.65
	AdaBoost	0.78	0.71	0.70	0.71	0.77	0.63
	XGB	0.80	0.75	0.68	0.70	0.79	0.64
	**F3 - Boruta Selected Feature**						
	**Model**	**Accuracy**	**Precision**	**Recall**	**F1 Score**	**AUC Score**	**AUPRC**
**Non ensemble Model**	CART	0.71	0.63	0.62	0.62	0.67	0.44
	SVM	0.71	0.63	0.63	0.63	0.66	0.42
	NB	0.67	0.61	0.63	0.62	0.64	0.39
	KNN	0.71	0.61	0.59	0.59	0.61	0.35
**Ensemble Model**	RF	0.73	0.65	0.63	0.63	0.69	0.47
	GBM	0.75	0.66	0.62	0.63	0.69	0.47
	AdaBoost	0.73	0.65	0.63	0.64	0.69	0.47
	XGB	0.74	0.65	0.61	0.62	0.68	0.46

The close agreement between cross-validation and test results suggests that the models are stable and do not exhibit substantial overfitting. Higher AUPRC values for F2-based models further indicate improved performance in handling class imbalance.

### Interpretation of McNemar’s test results

McNemar’s test was performed to examine whether the differences in predictive performance between model pairs were statistically significant under different feature selection methods.

### S5 Table (Lasso features-based models)

From S5 Table, it can be observed that most pairwise comparisons are **not statistically significant**, particularly among SVM, Random Forest, GBM, XGBoost, and AdaBoost, indicating comparable predictive performance. However, **Naïve Bayes exhibits highly significant differences** (p < 0.001) with the majority of models, suggesting inconsistent performance. Additionally, CART shows significant differences with several models, reflecting variability in its predictions.

### S6 Table (Chi-square features-based models)

From S6 Table, it is evident that a large proportion of comparisons are **statistically significant**, indicating substantial variability among model performances. CART differs significantly from most models except AdaBoost, while SVM and ensemble models also show significant differences in multiple comparisons. This suggests that the Chi-square feature selection method leads to more distinct model behaviors.

### S7 Table (Boruta features -based models)

From S7 Table, it can be seen that the results demonstrate a **moderate level of statistical significance**. Ensemble models frequently show significant differences when compared to simpler models such as CART, SVM, and Naïve Bayes, indicating improved predictive stability. However, several comparisons among ensemble models are not statistically significant, suggesting similar performance levels.

### Overall summary

Overall, from S5–S7 Tables, it can be concluded that **Naïve Bayes consistently shows significant differences** compared to other models, indicating less stable performance. In contrast, **ensemble** models demonstrate relatively consistent and robust performance, as reflected by the non-significant differences in many pairwise comparisons. Among the feature selection methods, **Chi-square** (S6 Table) shows the highest variability, whereas **Lasso** (S5 Table) indicates greater consistency across models.

### Recommended best feature selection technique

Out of the three feature selection methods, the **Chi-Square (F2)** approach demonstrates the best overall performance. It consistently achieves higher values across key evaluation metrics such as accuracy, F1-score, AUC, and AUPRC for multiple models, particularly for both non-ensemble and ensemble techniques. For instance, the **GBM** and **XGB** models using Chi-Square selected features attain the highest accuracy (**0.80**) and AUC (**0.79**), along with strong AUPRC values (up to **0.65**), indicating improved predictive performance and better class discrimination. Similarly, the **Random Forest** model achieves an F1-score of **0.71**, reflecting a balanced trade-off between precision and recall.

The superiority of the Chi-Square method can be attributed to its ability to identify statistically significant associations between categorical predictors and the outcome variable. This is particularly suitable for structured survey datasets such as BDHS, where many variables are categorical in nature. By selecting the most relevant features, the Chi-Square method reduces redundancy and enhances model interpretability while maintaining strong predictive performance.

In comparison, the LASSO (F1) method provides moderate performance, whereas the Boruta (F3) approach shows comparatively lower accuracy, AUC, and AUPRC across most models. Therefore, the results suggest that the Chi-Square feature selection method is a more effective and reliable approach for predicting risk in large-scale demographic datasets.

### Recommended best machine learning model

Based on the evaluation results presented in Table 5, the optimal models for each feature selection technique are as follows:

- **Lasso-selected features (F1):** Both CART and SVM achieved the highest F1-score (0.69), with CART slightly outperforming in accuracy (0.77) and maintaining competitive AUC (0.74) and AUPRC (0.54). Therefore, **CART** is the most suitable model for the Lasso feature set. Its classification performance is illustrated in the confusion matrix (S11 Fig).
- **Chi-square-selected features (F2): GBM** achieved the highest F1-score (0.71) along with strong accuracy (0.80), superior AUC (0.79), and robust AUPRC (0.64), making it the preferred model for this feature set. The corresponding confusion matrix is shown in (S12 Fig)
- **Boruta-selected features (F3): GBM** also demonstrated the best performance for Boruta-selected features, achieving slightly higher accuracy (0.75) while maintaining a good balance between precision and recall (F1-score 0.64, AUC 0.69). The confusion matrix for GBM on this feature set is presented in (S13 Fig)

**Overall**, CART (Lasso) and GBM (Chi-square and Boruta) emerged as the best-performing models for their respective feature selection techniques, and their confusion matrices provide a detailed view of class-wise prediction performance.

## Discussion

The results indicate that model performance is strongly influenced by the choice of feature selection method. Among the three approaches, the **Chi-square (F2)** feature set consistently achieved the best performance across models, particularly with ensemble methods. The **GBM** model showed the highest accuracy (0.80), AUC (0.79), and AUPRC (0.65), while **Random Forest** and **AdaBoost** also achieved strong F1-scores (0.71), indicating a good balance between precision and recall. In comparison, **Lasso (F1)** provided moderate performance, with **CART and SVM** achieving the highest F1-score (0.69) but lower AUPRC values. The **Boruta (F3)** method showed comparatively weaker results across all models, suggesting limited predictive improvement. Overall, ensemble models combined with Chi-square feature selection provide the most effective predictive performance, highlighting the importance of appropriate feature selection in improving model accuracy and discrimination.

Among the top variables, early marriage (Highest importance among actual features), current contraceptive method, occupation of husband, number of children, husband age, husband education, respondent current age and division (capturing regional disparities) emerged as the strongest risk factors. Regional disparities in pregnancy risk across Bangladesh appear to reflect differences in socioeconomic conditions, healthcare access, and cultural practices. Higher risk in Rangpur and Mymensingh reflects lower education, early marriage, and limited maternal care, while Dhaka benefits from better infrastructure and literacy. Education is a key protective factor, and husband’s age and education highlight the role of household decision making in maternal health.

The determinants identified here align closely with a broad body of DHS‑based investigations across South Asia and other low‑ and middle‑income countries (LMICs). These findings echo recent BDHS‑2022 analyses that identified age at first birth and education as primary LBW determinants [37], wealth and ANC visits as key C‑section predictors [38], and regional disparities in under‑5 mortality [39]. They also align with broader South‑Asian evidence linking early marriage, poverty and limited health‑service access to premature childbearing [40, 41]. Where prior work reported weaker effects of partner education, our model amplified its importance, perhaps because the chi‑square pre‑filter highlighted interaction‑rich variables that traditional regressions overlook.

A notable departure from some earlier Bangladeshi reports is the prominence of partner’s education and media exposure as independent risk factors. Earlier BDHS analyses [42] reported weaker associations for male education after adjusting for household wealth. The stronger signal observed in our chi‑square‑filtered model may stem from the filter’s capacity to capture univariate categorical relationships that are diluted in multivariable regressions. Similarly, Media exposure (newspaper, radio, television) has been inconsistently linked to contraceptive use and rarely to high-risk age at childbirth. However, this finding suggests that access to information or its absence directly influences reproductive timing decisions in rural Bangladesh.

The divergence underscores a methodological insight that conventional logistic regression may under‑estimate the contribution of variables that exhibit non‑linear or interaction effects (e.g., the joint impact of partner education and wealth). Tree‑based ensembles, by construction, uncover such patterns without the need for explicit interaction terms, thereby revealing determinants that are otherwise masked.

The BDHS dataset is dominated by categorical indicators (education level, wealth quintile, division). Chi‑square directly measures the association between each categorical predictor and the binary outcome, preserving information that Lasso may shrink or discard due to its reliance on continuous coefficient estimation. While Lasso mitigates multicollinearity through regularization, it can inadvertently penalize correlated predictors that are jointly informative (e.g., maternal and partner education). Chi‑square evaluates each variable independently, ensuring that all strong univariate signals are retained for the downstream model. The filter approach requires only one pass over the data, avoiding the iterative resampling and tree‑building steps inherent to Boruta, which can be unstable in high‑dimensional, sparsely populated DHS tables.

This study addressed confounding using multivariable logistic regression. However, some limitations remain. Complete case analysis may introduce selection bias due to missing data. Self-reported survey data may lead to recall and reporting bias. Residual confounding may still exist since some contextual factors like cultural norms and healthcare quality were not measured. Despite this, the nationally representative data supports generalizability.

The primary aim of the study was to “identify the key determinants of risk of birth in Bangladesh using BDHS 2022 data and provide evidence‑based insights on socio‑demographic and behavioral factors.” The high discriminatory performance of the chi‑square‑filtered RF model (AUC ≈ 0.79, accuracy ≈ 0.79) demonstrates that the selected predictors not only explain but also predict high-risk age at childbirth with practical relevance. Consequently, the objective has been met. We have distilled a parsimonious, empirically validated set of risk factors and demonstrated their predictive utility.

## Limitations

One important limitation of this study is the exclusion of observations with missing values, which resulted in a substantial reduction in sample size. This complete-case analysis approach may affect the national representativeness of the findings and introduce potential selection bias. Although a comparison between included and excluded observations suggested no major systematic differences, the possibility of residual bias cannot be entirely ruled out. Future studies should consider applying advanced missing data techniques, such as multiple imputation, to better preserve sample size and improve generalizability.

## Conclusion

By applying a chi‑square‑based feature selection and a random‑forest classifier to the BDHS 2022 data, we have demonstrated that high-risk birth in Bangladesh is driven by a synergistic constellation of young maternal age, current contraceptive method, occupation of husband, number of children, husband age, husband education, respondent current age and addressing regional disparities. These determinants are largely consistent with the broader South‑Asian and LMIC literature, yet the heightened relevance of partner education and media access underscores the added value of machine‑learning approaches for uncovering nuanced risk patterns. The findings substantiate the study’s objective and provide a robust, actionable evidence base for policymakers seeking to design targeted, multi‑sectoral strategies to delay first births and improve maternal‑child health in Bangladesh.

Policy‑relevant implications are delaying age at first birth through enforced minimum‑marriage ages, expanding female education, and improving facility‑based delivery in high‑risk districts could substantially curb high-risk age at childbirth. Targeted cash‑transfer schemes for the poorest households may also mitigate the wealth gradient. Collectively, these interventions align with the Sustainable Development Goal 3 target of reducing adolescent birth rates and improving maternal‑child health outcomes.

In sum, the chi‑square‑filtered random‑forest model provides an evidence‑based decision‑support tool for Bangladesh’s maternal‑health strategies, extending and refining the existing DHS literature.

## Supporting information

S1 Fig Prevalence of pregnancy risk across divisions of Bangladesh with 95% confidence intervals, based on BDHS 2022 data (N = 15,386). (TIF)

S2 Fig McNemar’s test p-value heatmap (Lasso), highlighting significant model differences (α = 0.05). (TIF)

S3 Fig McNemar’s test p-value heatmap (Chi-square), highlighting significant model differences (α = 0.05). (TIF)

S4 Fig McNemar’s test p-value heatmap (Boruta), highlighting significant model differences (α = 0.05). (TIF)

S5 Fig PRC curve of ensemble and non-ensemble machine learning models using Lasso features. (TIF)

S6 Fig PRC curve of ensemble and non-ensemble machine learning models using Chi square features. (TIF)

S7 Fig PRC curve of ensemble and non-ensemble machine learning models using Boruta features. (TIF)

S8 Fig Confusion Matrix for all ML using Lasso features. (TIF)

S9 Fig Confusion Matrix for all ML using Chi-square features. (TIF)

S10 Fig Confusion Matrix for all ML using Boruta features. (TIF)

S11 Fig Confusion Matrix of all Best model(CART) using Lasso features. (TIF)

S12 Fig Confusion Matrix of all Best model(GBM) using Chi-square features. (TIF)

S13 Fig Confusion Matrix of all Best model (GBM) using Boruta features. (TIF)

S1 Table Distribution of included vs. excluded participants across study variables (*p < 0.001; ns = not significant). (DOCX)

S2 Table Prevalence of pregnancy risk in the study sample with 95% Confidence Interval. (DOCX)

S3 Table Prevalence of Pregnancy Risk with 95% Confidence Intervals. (DOCX)

S4 Table Adjusted Odds Ratios (AOR) from Logistic Regression Analysis. (DOCX)

S5 Table Mc Nayem’s Table using Lasso selected Feature. (DOCX)

S6 Table Mc Nayem’s Table using Chi-square selected Feature. (DOCX)

S7 Table Mc Nayem’s Table using Boruta selected Features. (DOCX)
