# Supplementary material for: Uncovering the determinants of high-risk age at childbirth in Bangladesh: A machine learning analysis of the BDHS 2022 data
Source: PLoS One. 2026 Aug 20;21(8):e0356362. doi: 10.1371/journal.pone.0356362 (PMC13492789; doi:10.1371/journal.pone.0356362)
Supplement: Supplementary file 1 [file pone.0356362.s014.docx]

**S1 Table. Distribution of included vs. excluded participants across study variables (*p < 0.001; ns = not significant).**

| Variable | Included n (%) | Excluded n (%) | p-value | Significance |
| --- | --- | --- | --- | --- |
| Place of Residence | 10,080 (65.5%) | 375 (57.5%) | <0.001 | *** |
| Division | 2,352 (15.3%) | 90 (13.8%) | <0.001 | *** |
| Highest Educational Level | 7,004 (45.5%) | 369 (56.6%) | <0.001 | *** |
| Religion | 13,727 (89.2%) | 629 (96.5%) | <0.001 | *** |
| Literacy | 9,541 (62.0%) | 544 (83.4%) | <0.001 | *** |
| Wealth Index | 3,262 (21.2%) | 247 (37.9%) | <0.001 | *** |
| Husband Desire for Children | 13,409 (87.2%) | 648 (99.4%) | <0.001 | *** |
| Exposure | 11,167 (72.6%) | 600 (92.0%) | <0.001 | *** |
| Husband Education Level | 4,855 (31.6%) | 229 (35.1%) | <0.001 | *** |
| Respondent Currently Working | 10,515 (68.3%) | 506 (77.6%) | <0.001 | *** |
| Respondent Current Age | 3,379 (22.0%) | 186 (28.5%) | <0.001 | *** |
| Husband Age | 8,566 (55.7%) | 429 (65.8%) | <0.001 | *** |
| Number of Children | 11,324 (73.6%) | 589 (90.3%) | <0.001 | *** |
| Occupation of Husband | 6,560 (42.6%) | 254 (39.0%) | <0.001 | *** |
| Contraceptive Method | 8,443 (54.9%) | 536 (82.2%) | <0.001 | *** |
| Early Marriage | 12,051 (78.3%) | 500 (76.7%) | 0.345 | ns |
| Risk of Pregnancy (Target) | 4,099 (26.6%) | 102 (15.6%) | <0.001 | *** |
