# Supplementary material for: Uncovering the determinants of high-risk age at childbirth in Bangladesh: A machine learning analysis of the BDHS 2022 data
Source: PLoS One. 2026 Aug 20;21(8):e0356362. doi: 10.1371/journal.pone.0356362 (PMC13492789; doi:10.1371/journal.pone.0356362)
Supplement: Supplementary file 2 [file pone.0356362.s015.docx]

**S2 Table:** Prevalence of pregnancy risk in the study sample with 95% **Confidence Interval.**

| Indicator | Value |
| --- | --- |
| Total Sample (n) | 15,386 |
| At Risk | 4,099 |
| Prevalence (%) | 26.60% |
| 95% Confidence Interval | 25.9% – 27.3% |
