# Supplementary material for: Uncovering the determinants of high-risk age at childbirth in Bangladesh: A machine learning analysis of the BDHS 2022 data
Source: PLoS One. 2026 Aug 20;21(8):e0356362. doi: 10.1371/journal.pone.0356362 (PMC13492789; doi:10.1371/journal.pone.0356362)
Supplement: Supplementary file 3 [file pone.0356362.s016.docx]

**S3 Table.** Prevalence of Pregnancy Risk with 95% **Confidence Intervals.**

| Variables | Categories | n | At Risk | % | 95% CI |
| --- | --- | --- | --- | --- | --- |
| Division | Chittagong | 1,695 | 449 | 26.50% | 24.4% – 28.6% |
|  | Dhaka | 2,352 | 552 | 23.50% | 21.8% – 25.2% |
|  | Khulna | 2,282 | 583 | 25.50% | 23.8% – 27.4% |
|  | Mymensingh | 1,979 | 590 | 29.80% | 27.8% – 31.9% |
|  | Rajshahi | 1,694 | 441 | 26.00% | 24.0% – 28.2% |
|  | Rangpur | 1,903 | 567 | 29.80% | 27.8% – 31.9% |
|  | Sylhet | 1,855 | 512 | 27.60% | 25.6% – 29.7% |
| Education Level | No Education | 2,025 | 1,162 | 57.40% | 55.2% – 59.5% |
|  | Primary | 4,253 | 1,285 | 30.20% | 28.9% – 31.6% |
|  | Secondary | 7,004 | 1,303 | 18.60% | 17.7% – 19.5% |
|  | Higher | 2,104 | 349 | 16.60% | 15.1% – 18.2% |
| Residence | Rural | 5,306 | 1,416 | 26.70% | 25.5% – 27.9% |
| Wealth Category | Poorer | 2,797 | 680 | 24.30% | 22.8% – 25.9% |
|  | Middle | 3,054 | 810 | 26.50% | 25.0% – 28.1% |
|  | Richer | 3,103 | 829 | 26.70% | 25.2% – 28.3% |
|  | Richest | 3,170 | 830 | 26.20% | 24.7% – 27.7% |
