# Supplementary material for: Uncovering the determinants of high-risk age at childbirth in Bangladesh: A machine learning analysis of the BDHS 2022 data
Source: PLoS One. 2026 Aug 20;21(8):e0356362. doi: 10.1371/journal.pone.0356362 (PMC13492789; doi:10.1371/journal.pone.0356362)
Supplement: Supplementary file 4 [file pone.0356362.s017.docx]

**S4 Table.** Adjusted Odds Ratios (AOR) from Logistic Regression Analysis

| Feature | Adjusted OR | 95% Confidence Interval | Interpretation |
| --- | --- | --- | --- |
| Husband education level | 1.09 | 1.044 – 1.139 | ↑ Risk factor |
| Number of children | 1.136 | 1.066 – 1.211 | ↑ Risk factor |
| Literacy | 0.751 | 0.651 – 0.867 | ↓ Protective |
| Type of place of residence | 0.89 | 0.840 – 0.943 | ↓ Protective |
| Highest educational level | 0.658 | 0.534 – 0.811 | ↓ Protective |
| Exposure | 1.549 | 1.245 – 1.928 | ↑ Risk factor |
| Early marriage | 1.001 | 1.000 – 1.001 | ↑ Risk factor |
| Husband age | 1.993 | 1.412 – 2.813 | ↑ Risk factor |
| Respondent currently working | 1.053 | 1.026 – 1.080 | ↑ Risk factor |
