# Supplementary material for: Uncovering the determinants of high-risk age at childbirth in Bangladesh: A machine learning analysis of the BDHS 2022 data
Source: PLoS One. 2026 Aug 20;21(8):e0356362. doi: 10.1371/journal.pone.0356362 (PMC13492789; doi:10.1371/journal.pone.0356362)
Supplement: Supplementary file 5 [file pone.0356362.s018.docx]

**S5 Table.** Mc Nayem’s Table using Lasso selected Features

| Model A | Model B | b | c | p-value | Significance Level |
| --- | --- | --- | --- | --- | --- |
| CART | SVM | 33 | 23 | 0.2291 | Not Significant |
| CART | Naive Bayes | 489 | 200 | 0 | p<0.001 Highly Significant |
| CART | KNN | 145 | 107 | 0.0198 | p<0.05 Significant |
| CART | Random Forest | 26 | 11 | 0.0214 | p<0.05 Significant |
| CART | GBM | 28 | 16 | 0.0973 | Not Significant |
| CART | XGB | 31 | 18 | 0.0865 | Not Significant |
| CART | AdaBoost | 84 | 51 | 0.0059 | p<0.01 Very Significant |
| SVM | Naive Bayes | 466 | 187 | 0 | p<0.001 Highly Significant |
| SVM | KNN | 160 | 132 | 0.1141 | Not Significant |
| SVM | Random Forest | 28 | 23 | 0.5754 | Not Significant |
| SVM | GBM | 26 | 24 | 0.8875 | Not Significant |
| SVM | XGB | 26 | 23 | 0.7751 | Not Significant |
| SVM | AdaBoost | 93 | 70 | 0.0849 | Not Significant |
| Naive Bayes | KNN | 268 | 519 | 0 | p<0.001 Highly Significant |
| Naive Bayes | Random Forest | 204 | 478 | 0 | p<0.001 Highly Significant |
| Naive Bayes | GBM | 206 | 483 | 0 | p<0.001 Highly Significant |
| Naive Bayes | XGB | 204 | 480 | 0 | p<0.001 Highly Significant |
| Naive Bayes | AdaBoost | 231 | 487 | 0 | p<0.001 Highly Significant |
| KNN | Random Forest | 129 | 152 | 0.1894 | Not Significant |
| KNN | GBM | 125 | 151 | 0.1324 | Not Significant |
| KNN | XGB | 130 | 155 | 0.1551 | Not Significant |
| KNN | AdaBoost | 146 | 151 | 0.8165 | Not Significant |
| Random Forest | GBM | 12 | 15 | 0.7003 | Not Significant |
| Random Forest | XGB | 11 | 13 | 0.8388 | Not Significant |
| Random Forest | AdaBoost | 69 | 51 | 0.1207 | Not Significant |
| GBM | XGB | 5 | 4 | 1 | Not Significant |
| GBM | AdaBoost | 76 | 55 | 0.0806 | Not Significant |
| XGB | AdaBoost | 79 | 59 | 0.1058 | Not Significant |
