# Supplementary material for: Uncovering the determinants of high-risk age at childbirth in Bangladesh: A machine learning analysis of the BDHS 2022 data
Source: PLoS One. 2026 Aug 20;21(8):e0356362. doi: 10.1371/journal.pone.0356362 (PMC13492789; doi:10.1371/journal.pone.0356362)
Supplement: Supplementary file 6 [file pone.0356362.s019.docx]

**S6** Table. Mc Nayem’s Table using Chi-square selected Features

| Model A | Model B | b | c | p-value | Significance Level |
| --- | --- | --- | --- | --- | --- |
| CART | SVM | 247 | 148 | 0 | p<0.001 Highly Significant |
| CART | Naive Bayes | 315 | 169 | 0 | p<0.001 Highly Significant |
| CART | KNN | 402 | 198 | 0 | p<0.001 Highly Significant |
| CART | Random Forest | 95 | 140 | 0.0041 | p<0.01 Very Significant |
| CART | GBM | 102 | 150 | 0.0031 | p<0.01 Very Significant |
| CART | XGB | 101 | 153 | 0.0014 | p<0.01 Very Significant |
| CART | AdaBoost | 161 | 149 | 0.5321 | Not Significant |
| SVM | Naive Bayes | 340 | 293 | 0.0675 | Not Significant |
| SVM | KNN | 289 | 184 | 0 | p<0.001 Highly Significant |
| SVM | Random Forest | 96 | 240 | 0 | p<0.001 Highly Significant |
| SVM | GBM | 96 | 243 | 0 | p<0.001 Highly Significant |
| SVM | XGB | 89 | 240 | 0 | p<0.001 Highly Significant |
| SVM | AdaBoost | 165 | 252 | 0 | p<0.001 Highly Significant |
| Naive Bayes | KNN | 357 | 299 | 0.026 | p<0.05 Significant |
| Naive Bayes | Random Forest | 131 | 322 | 0 | p<0.001 Highly Significant |
| Naive Bayes | GBM | 151 | 345 | 0 | p<0.001 Highly Significant |
| Naive Bayes | XGB | 156 | 354 | 0 | p<0.001 Highly Significant |
| Naive Bayes | AdaBoost | 146 | 280 | 0 | p<0.001 Highly Significant |
| KNN | Random Forest | 139 | 388 | 0 | p<0.001 Highly Significant |
| KNN | GBM | 134 | 386 | 0 | p<0.001 Highly Significant |
| KNN | XGB | 131 | 387 | 0 | p<0.001 Highly Significant |
| KNN | AdaBoost | 175 | 367 | 0 | p<0.001 Highly Significant |
| Random Forest | GBM | 60 | 63 | 0.8569 | Not Significant |
| Random Forest | XGB | 68 | 75 | 0.6158 | Not Significant |
| Random Forest | AdaBoost | 122 | 65 | 0 | p<0.001 Highly Significant |
| GBM | XGB | 26 | 30 | 0.6885 | Not Significant |
| GBM | AdaBoost | 158 | 98 | 0.0002 | p<0.001 Highly Significant |
| XGB | AdaBoost | 174 | 110 | 0.0002 | p<0.001 Highly Significant |
