# Supplementary material for: Uncovering the determinants of high-risk age at childbirth in Bangladesh: A machine learning analysis of the BDHS 2022 data
Source: PLoS One. 2026 Aug 20;21(8):e0356362. doi: 10.1371/journal.pone.0356362 (PMC13492789; doi:10.1371/journal.pone.0356362)
Supplement: Supplementary file 7 [file pone.0356362.s020.docx]

**S7 Table.** Mc Nayem’s Table using Boruta selected Features

| Model A | Model B | b | c | p-value | Significance Level |
| --- | --- | --- | --- | --- | --- |
| CART | SVM | 169 | 159 | 0.6192 | Not Significant |
| CART | Naive Bayes | 323 | 190 | 0 | p<0.001 Highly Significant |
| CART | KNN | 259 | 250 | 0.7229 | Not Significant |
| CART | Random Forest | 109 | 162 | 0.0016 | p<0.01 Very Significant |
| CART | GBM | 107 | 207 | 0 | p<0.001 Highly Significant |
| CART | XGB | 125 | 196 | 0.0001 | p<0.001 Highly Significant |
| CART | AdaBoost | 166 | 225 | 0.0034 | p<0.01 Very Significant |
| SVM | Naive Bayes | 336 | 213 | 0 | p<0.001 Highly Significant |
| SVM | KNN | 247 | 248 | 1 | Not Significant |
| SVM | Random Forest | 76 | 139 | 0 | p<0.001 Highly Significant |
| SVM | GBM | 108 | 218 | 0 | p<0.001 Highly Significant |
| SVM | XGB | 129 | 210 | 0 | p<0.001 Highly Significant |
| SVM | AdaBoost | 134 | 203 | 0.0002 | p<0.001 Highly Significant |
| Naive Bayes | KNN | 323 | 447 | 0 | p<0.001 Highly Significant |
| Naive Bayes | Random Forest | 172 | 358 | 0 | p<0.001 Highly Significant |
| Naive Bayes | GBM | 213 | 446 | 0 | p<0.001 Highly Significant |
| Naive Bayes | XGB | 242 | 446 | 0 | p<0.001 Highly Significant |
| Naive Bayes | AdaBoost | 130 | 322 | 0 | p<0.001 Highly Significant |
| KNN | Random Forest | 195 | 257 | 0.0041 | p<0.01 Very Significant |
| KNN | GBM | 151 | 260 | 0 | p<0.001 Highly Significant |
| KNN | XGB | 169 | 249 | 0.0001 | p<0.001 Highly Significant |
| KNN | AdaBoost | 225 | 293 | 0.0032 | p<0.01 Very Significant |
| Random Forest | GBM | 69 | 116 | 0.0007 | p<0.001 Highly Significant |
| Random Forest | XGB | 98 | 116 | 0.2452 | Not Significant |
| Random Forest | AdaBoost | 99 | 105 | 0.7263 | Not Significant |
| GBM | XGB | 68 | 39 | 0.0068 | p<0.01 Very Significant |
| GBM | AdaBoost | 152 | 111 | 0.0136 | p<0.05 Significant |
| XGB | AdaBoost | 170 | 158 | 0.5436 | Not Significant |
