# Supplementary material for: Uncovering the determinants of high-risk age at childbirth in Bangladesh: A machine learning analysis of the BDHS 2022 data
Source: PLoS One. 2026 Aug 20;21(8):e0356362. doi: 10.1371/journal.pone.0356362 (PMC13492789; doi:10.1371/journal.pone.0356362)
Supplement: Supplementary file 10 [file pone.0356362.s024.docx]

**Additional Editor Comments:**

**1. The most important remaining issue is outcome inconsistency. The manuscript title and narrative emphasize early pregnancy/early childbirth, but the Methods define the outcome as high-risk age at childbirth, coded as high risk for ≤18 years or ≥40 years and low risk for 19–39 years. This changes the scientific meaning of the study and must be harmonized before acceptance**.

**Response:** Thank you for this important observation. We agree that the terminology used in the previous version of the manuscript created inconsistency between the study narrative and the outcome variable definition. The outcome analyzed in this study was maternal age at childbirth, categorized as high-risk (≤18 years or ≥40 years) and low-risk (19–39 years). Therefore, the term “high-risk age at childbirth” more accurately reflects the outcome investigated than “early pregnancy” or “early childbirth.”

To address this concern, we have thoroughly revised the manuscript to harmonize the terminology throughout all sections. Specifically, references to “early pregnancy” and “early childbirth” have been replaced with “high-risk age at childbirth” in the title, abstract, introduction, results, discussion, tables, and supplementary materials where appropriate. We have also clarified the operational definition of the outcome variable in the Methods section to ensure consistency between the study objective, outcome definition, analytical approach, and interpretation of findings.

These revisions align the manuscript with the actual outcome analyzed and eliminate the inconsistency identified by the reviewer.

**2. The sample derivation and missing-data explanation remain vulnerable. Excluding 14,040 observations is substantial, and the response should not overstate representativeness or claim minimal bias if many included/excluded variables differ significantly.**

**Response:** Thank you for this important comment. We agree that the exclusion of 14,040 observations due to missing data is substantial and warrants careful consideration regarding potential selection bias and representativeness.

To address this concern, we compared the characteristics of included and excluded observations and found statistically significant differences for several variables. Accordingly, we have revised the manuscript to avoid overstating the representativeness of the final analytic sample or suggesting that the exclusions introduced only minimal bias. Instead, we explicitly acknowledge that these differences may have affected the composition of the study sample and could limit the generalizability of the findings.

In addition, we conducted further analyses to assess the potential impact of sample exclusion and have reported the corresponding results in the revised manuscript. While these analyses provide additional insight into the extent of potential bias, they cannot completely eliminate concerns arising from missing data and sample reduction.

To ensure transparency, we have expanded the description of sample derivation in the Methods section and added a dedicated discussion in the Limitations section. We now explicitly state that the complete-case analysis may be subject to selection bias because excluded observations differed from included observations on several characteristics. Therefore, the findings should be interpreted with appropriate caution and may not be fully representative of the entire target population.

**3. The best-performing model is not consistently reported across the abstract, results, response letter, and tables. This should be corrected by specifying whether the “best” model is based on cross-validation, final test-set performance, AUROC, AUPRC, F1, or accuracy.**

**Response:** Thank you for highlighting this inconsistency. To improve clarity and ensure consistent reporting throughout the manuscript, we have revised the abstract, results section, tables, and response letter to explicitly state that model performance was evaluated using six metrics: accuracy, precision, recall, F1-score, AUROC, and AUPRC. While all six metrics were considered in assessing model performance, AUROC was used as the primary reference metric for evaluating overall discriminatory ability.

Based on the combined evaluation of these performance measures, CART (using Lasso-selected features) and GBM (using Chi-square- and Boruta-selected features) demonstrated the strongest overall performance within their respective feature-selection approaches. In addition, the revised abstract now explicitly reports the best-performing model(s) together with their corresponding performance metrics (accuracy, precision, recall, F1-score, AUROC, and AUPRC) to provide a clear and transparent summary of model performance. We have ensured that the identification and reporting of the best-performing models are consistent across the abstract, results, tables, and supplementary materials.

**Review Comments to the Author**

**Reviewer #1: The author has made improvements according to the input I provided very well, including improvements to the abstract, introduction, methods, results, discussion and conclusions. There are no more objections from me.**

**Response:** Thank you for your positive assessment of our revised manuscript. We appreciate your careful review and valuable suggestions, which helped improve the quality, clarity, and overall presentation of the study. We are pleased that the revisions have satisfactorily addressed your concerns.

**Reviewer #2: The authors appear to have made a reasonable effort to address the concerns raised by the reviewers. However, several of the revisions seem primarily aimed at providing justifications in response to reviewer comments rather than fully integrating the corresponding changes into the manuscript itself. Furthermore, the manuscript does not consistently adhere to the formatting requirements and guidelines of PLOS. The authors are encouraged to carefully review the journal's formatting policies and ensure full compliance prior to resubmission.**

**Response:** Thank you for your thoughtful comments and for recognizing our efforts to address the concerns raised during the review process. We appreciate your observation that some revisions may have been more evident in the response letter than in the manuscript itself.

In response, we have carefully reviewed the entire manuscript and ensured that all substantive revisions described in the response letter have been fully incorporated into the manuscript text where appropriate. This includes revisions to the title, abstract, introduction, methods, results, discussion, limitations, and conclusions to ensure consistency, clarity, and transparency throughout the paper.

Additionally, we have conducted a thorough review of the manuscript to ensure compliance with PLOS ONE formatting requirements and submission guidelines. We have revised the manuscript accordingly, including formatting, section organization, figure and table presentation, references, and supplementary materials, to align with the journal’s requirements.

We appreciate this important recommendation and believe that these additional revisions have strengthened both the scientific content and the presentation of the manuscript.

**Reviewer #3: All of the concerns appear to have been adequately addressed by the authors. I am confident that this review meets the standards required by PLOS One.**

**Response:** Thank you for your encouraging comments and positive evaluation of our manuscript. We appreciate your careful review and are grateful for your assessment that the concerns raised during the review process have been adequately addressed. Your feedback has been invaluable in improving the quality of our work.
