# Supplementary material for: Uncovering the determinants of high-risk age at childbirth in Bangladesh: A machine learning analysis of the BDHS 2022 data
Source: PLoS One. 2026 Aug 20;21(8):e0356362. doi: 10.1371/journal.pone.0356362 (PMC13492789; doi:10.1371/journal.pone.0356362)
Supplement: Supplementary file 11 [file pone.0356362.s022.docx]

Editor’s Response

**1. Abstract**

The abstract is clear and includes data source, outcome, methods, and key findings, but the framing can be sharper and more neutral. It should more explicitly specify study design and sample characteristics and avoid promotional wording to align with PLOS ONE’s scientific tone. It is advisable to replace phrases like “key ingredients” or “exceptionally effective” with neutral alternatives such as “most influential factors” and “showed the highest predictive performance,” and add a phrase such as “cross-sectional secondary analysis of 8,000 ever-married women from BDHS 2022.”

**Response:** Thank you for your constructive feedback. We have revised the Abstract to improve clarity, precision, and adherence to a neutral scientific tone consistent with PLOS ONE guidelines.

Specifically, we now explicitly state the study design and sample characteristics, describing the analysis as a cross-sectional secondary analysis of BDHS 2022 data and clearly reporting the study population. In addition, we have replaced informal or promotional wording (e.g., “key ingredients,” “exceptionally effective”) with more neutral scientific expressions. These revisions enhance the clarity, transparency, and academic rigor of the abstract.

**2. Introduction**

The Introduction is informative but suffers from repetition and does not clearly converge on the specific knowledge gap and novelty. Adverse outcomes and general determinants (education, poverty, rural residence) are reiterated several times, while the unique contribution—using BDHS 2022 with ML model comparison—is underemphasized. It is advisable to restructure the section into four paragraphs (global burden → Bangladesh context → known determinants → explicit gap and objectives) and add a clear gap sentence such as: “No previous study has systematically compared machine learning algorithms to predict early first birth using BDHS 2022 data.”

Some language in the Introduction is informal or imprecise for a scientific article. Informal terms (“first kid”, “big effect”) and vague phrasing reduce the perceived rigor of the manuscript. It is advisable to use more formal phrasing like “first child,” “substantial impact,” and describe psychosocial consequences in precise terms, e.g., lower educational attainment and reduced economic opportunities.

**Response:** Thank you for your valuable comments on our manuscript. We have carefully revised the Introduction to address all concerns.

Specifically, we restructured it into four clear paragraphs (global context, Bangladesh context, determinants, and research gap), removed repetition, and improved the academic tone. We also explicitly clarified the study’s novelty by adding the gap statement: “No previous study has systematically compared machine learning algorithms to predict early first birth using BDHS 2022 data.”

We believe these revisions have improved the clarity and contribution of the manuscript. Thank you for your consideration.

**3. Methods**

1. Terminology for the main outcome is inconsistent, with references to “early marriage” instead of “early first birth” in several places. This inconsistency strongly suggests copy-paste from another project and can undermine reviewer confidence in the care taken with the analysis. **It is advisable to s**ystematically replace “early marriage” with “early first birth (≤19 years)” and ensure that all descriptions of classes, SMOTE, and the conceptual framework use the correct outcome term. [Done]

**Response:** Thank you for highlighting this important issue. We sincerely apologize for the inconsistency in terminology, which was unintentional and has now been carefully corrected throughout the manuscript.

In the revised version, we have updated and clarified the definition of the outcome variable. Specifically, the target variable has been redefined as **“risk at birth”**, constructed based on the respondent’s age at childbirth. The variable is coded as **1 (high risk)** for women who gave birth at ≤18 years or ≥40 years, and **0 (low risk/normal)** for those who gave birth between 19 and 39 years.

All instances of incorrect terminology, including “early marriage,” have been systematically replaced to ensure consistency across the manuscript, including in the descriptions of class labels, SMOTE application, and the conceptual framework. This clarification has been explicitly detailed in the **Methodology section under the “Target Variable” subsection**.

We appreciate the reviewer’s careful observation, which has helped improve the clarity and consistency of our study.

2. The derivation of the final analytic sample of 8,000 women is not fully transparent. A clear description of who was excluded and why (never-married, missing age at first birth, missing covariates, etc.) is needed to interpret the generalizability and risk of selection bias. **It is advisable to a**dd a concise sample-flow description (and ideally a simple diagram), such as: “From 16,038 interviewed women aged 15–49, we excluded never-married women, those without recorded age at first birth, and those with missing values in key covariates, yielding 8,000 ever-married women for analysis.” [Done]

**Response:** We included 16 independent predictors based on the literature review. Several variables contained missing values; however, no imputation techniques were applied, as prior evidence suggests that certain machine learning models may perform sub optimally with imputed data. The approach to handling missing data is described in the methodology section under ***“Sample Size and Handling of Missing Data.”*** Additionally, the process used to derive the final analytical sample is illustrated in **Fig 2** through a detailed sample flowchart.

3. The ML pipeline is generally described, but **model names, SMOTE usage, and software environment** are not always clearly specified.  Ambiguous abbreviations (e.g., “RM”), lack of detail on when SMOTE is applied, and missing information on software packages hinder reproducibility. **It is advisable to s**tandardize abbreviations (LR, CART, RF, GBM, XGB, KNN), explicitly state that SMOTE was applied within each training fold of cross-validation, and specify that analyses were conducted in Python with scikit-learn and XGBoost (with version numbers if possible). [Done]

**Response:**

Thank you for this valuable suggestion, which has helped us improve the clarity and reproducibility of our methodology.

In the revised manuscript, we have standardized all model abbreviations to commonly accepted forms, including Classification and Regression Trees (CART), Random Forest (RF), Gradient Boosting Machine (GBM), Adaptive Boosting (AdaBoost), Extreme Gradient Boosting (XGB), and K-Nearest Neighbors (KNN), Naïve Bayes (NB). Ambiguous abbreviations such as “RM” have been removed to avoid confusion.

We have also clarified the implementation of the SMOTE technique in the research design section. Specifically, SMOTE was applied **only to the training data**, ensuring that no information from the test data was used during model training and thereby preventing data leakage. This detail has now been explicitly stated in the Methods section.

Furthermore, we have added a detailed description of the software environment used for the analysis. All models were implemented in **Python**, using the scikit-learn library for LR, CART, RF, GBM, and KNN, and the XGB library for XGB. The corresponding version numbers have also been included in the revised manuscript to enhance reproducibility.

All experiments were conducted using Python (version 3.11) on the Google Colaboratory (Colab) platform, utilizing an NVIDIA T4 GPU, Intel Xeon 2.20 GHz CPU, and 12 GB RAM. Data preprocessing and manipulation were performed using Pandas (version 2.2.2) and NumPy (version 2.0.2), while visualization was conducted using Matplotlib (version 3.5.2) and Seaborn (version 0.12.2). Machine learning models, including Logistic Regression (LR), Classification and Regression Trees (CART), Random Forest (RF), Gradient Boosting Machine (GBM), Extreme Gradient Boosting (XGB), and K-Nearest Neighbors (KNN), were implemented using Scikit-learn (version 1.6.1) and XGB (version 2.0.2), with additional deep learning support from TensorFlow (version 2.12).

Model performance was evaluated using 10-fold cross-validation to ensure robust generalization. To address class imbalance, the Synthetic Minority Oversampling Technique (SMOTE) was applied using the imbalanced-learn package (version 0.14.1). Importantly, SMOTE was applied only to the training data within each cross-validation fold, thereby preventing data leakage and preserving the integrity of the test data.These revisions have been incorporated into the **Experimental Settings Results section** of the manuscript.

We appreciate the reviewer’s insightful comments, which have significantly strengthened the transparency and reproducibility of our study.

**4. Results**

 1. The narrative for the descriptive statistics is overly detailed and repetitive, listing many cell percentages from the table. This level of detail obscures the main patterns and makes the Results section longer and harder to follow. **It is advisable to c**ondense the description of Table 2 to highlight only the strongest gradients (education, wealth, rural vs urban residence, age at marriage, contraceptive use) in a short interpretive paragraph instead of enumerating many individual percentages. [Done]

**Response:** Thank you for this helpful suggestion. In response, we have revised the descriptive statistics section for Table 2 by removing repetitive reporting of individual cell percentages and condensing the narrative. The revised text now emphasizes the key gradients—education, wealth, rural–urban residence, age at marriage, and contraceptive use—through a concise and interpretive summary. This has improved the clarity and readability of the Results section.

2. Some narrative statements (e.g., about which divisions have the highest prevalence) do not perfectly align with the percentages in the tables. Any mismatch between text and tables is a technical inaccuracy that reviewers quickly notice and may question. **It is advisable to r**echeck and rewrite those sentences so they conform to the table, for instance: “Rangpur and Khulna show the highest prevalence of early birth, whereas Sylhet and Dhaka have comparatively lower levels,” if that matches the reported data. [Done]

**Response:**

Thank you for highlighting this issue. In the revised manuscript, Table 2 has been updated, and all corresponding narrative statements have been carefully rechecked and aligned with the reported percentages. The descriptions have been rewritten to ensure full consistency between the text and the table, minimizing the possibility of any discrepancies. We believe this revision has addressed the concern effectively.

3. Model performance and feature-selection results are described in lengthy and somewhat repetitive paragraphs. Repeating similar performance metrics for multiple models and feature-selection schemes reduces clarity and can overwhelm the reader. **It is advisable to p**rovide a synthesized comparison such as: “Across all feature-selection methods, CART, particularly when combined with Chi-square–selected features, yielded the highest overall performance, with GBM and RF performing similarly but not consistently outperforming CART.” [Done]

**Response:**

Thank you for this valuable suggestion. The primary objective of this study is to compare the performance of ensemble and non-ensemble models across three distinct feature-selection methods, namely Lasso (F1), Chi-square (F2), and Boruta (F3). As a result, model performance has been reported separately for each feature set to maintain methodological clarity and ensure a fair comparison.

However, we acknowledge that this approach may reduce readability. Accordingly, we have revised the Results section to minimize repetition and include a more synthesized summary of findings. In particular, we now clearly highlight that **CART** demonstrated the best performance with Lasso-selected features, whereas **GBM** consistently performed best with both Chi-square and Boruta feature sets. This revision improves clarity while preserving the comparative objective of the study.

5. Discussion and Conclusion

The Discussion appropriately revisits key findings but sometimes repeats background material instead of focusing on the interpretation of this study’s specific results. Discussion sections should synthesize how the current findings compare with previous work and what they imply, rather than re-explaining general facts about early childbearing. It is advisable to rephrase generic statements as comparative ones: “Our findings confirm and extend previous evidence that rural residence, lower wealth, and lack of formal education are strongly associated with early first birth in Bangladesh and other LMICs.” The added value of machine learning (especially CART and GBM) is not fully translated into practical or policy implications. Since the study compares ML models, reviewers will expect a clearer explanation of how these models could be used in targeting interventions or creating risk tools. It is advisable to add a paragraph explaining that CART trees can be converted into simple decision rules or risk scores based on combinations of education, age at marriage, and wealth, which frontline workers could use to identify adolescents at high risk of early first birth. The Conclusion includes some informal or conversational phrases that are not ideal for a scientific journal. A concise, neutral conclusion is more appropriate for PLOS ONE and reinforces the scientific tone. It is advisable to replace phrases such as “beyond the numbers” with a more neutral summary: “A small set of socio-demographic factors— particularly education, age at marriage, household wealth, and contraceptive use—largely determines the risk of first birth before age 19, and ML models like CART can help target prevention efforts.”

**Response:** Thank you for these valuable suggestions. We have revised both the **Discussion** and **Conclusion** sections to improve clarity, focus, and scientific tone.

First, the Discussion has been refined to reduce repetition of general background information and instead emphasize the interpretation of our study-specific findings. We have incorporated more **comparative statements** to align our results with existing literature, highlighting how our findings confirm and extend previous evidence on key determinants such as education, age at marriage, household wealth, and place of residence in Bangladesh and similar LMIC contexts.

Second, we have strengthened the **practical and policy implications** of the machine learning models. In particular, we added a paragraph explaining how models such as **CART** can be translated into **simple decision rules or risk stratification tools**, which can be applied by frontline health workers to identify adolescents at high risk of early first birth based on key socio-demographic characteristics. We also clarified the added value of ensemble models such as **GBM** in improving predictive accuracy for population-level targeting.

Finally, the **Conclusion** section has been revised to remove informal language and adopt a more concise, neutral, and scientific tone, in line with journal expectations. The revised conclusion now clearly summarizes that a limited set of socio-demographic factors—particularly education, age at marriage, household wealth, and contraceptive use—are strong predictors of early first birth, and that machine learning approaches can support more targeted prevention strategies.

These revisions improve the overall coherence, rigor, and applicability of the manuscript.

Reviewer 1 Response

**Abstract**:

**Please explain the research design, sample size, inclusion and exclusion criteria**

**Response:** Thank you for your suggestion. We have revised the Abstract to clearly describe the research design, sample size, and eligibility criteria. Specifically, we now state that the study is a cross-sectional secondary analysis of BDHS 2022 data, and we report the final sample size included in the analysis. We have also briefly outlined the inclusion criteria (ever-married women within the reproductive age group with complete information on study variables) and the exclusion criteria (cases with missing values and duplicate observations removed during data preprocessing).

These additions improve the transparency and completeness of the abstract while maintaining a concise scientific presentation.

**Introduction:**

**At the end of the paragraph in the introduction, please add the research objective**

**Response:** We have revised the Introduction to include a clear statement of the research objective at the end of the final paragraph.

Methods:

**1. Explain the research design [Done]**

**Response:** Thank you for the suggestion. The research design has now been clearly described in the Methodology section, and a detailed schematic representation has been added as Fig 1 to illustrate the overall study workflow.

**• Important Concerns on Data Pre-processing:**

**1) The authors excluded approximately 50% of the initial sample (from 16,038 to 8,000) due to missing information. This massive reduction raises significant concerns regarding Selection Bias. [Done]**

**Response:** The initial dataset comprised **30,077** observations. Several predictors selected from the literature contained missing values. As the objective of this study was to develop machine learning models using complete-case analysis, no imputation was performed; instead, observations with missing values were excluded. After removing cases with missing data, the sample size was reduced to 16,038. Subsequently, 652 duplicate records were identified and removed, resulting in a final analytical sample of 15,386 observations. Detailed information on sample selection and data cleaning procedures is provided in the Methodology section under ***“Sample Size and Handling of Missing Data”*** and illustrated in **Fig 2.**

As the work was to show the comparison machine learning model previously, I used sample data of the cleaned data 16,038. That was my limitation. I correct it in the updated manuscript.

**2) Authors should provide a comparison table between the excluded and included groups to show that there are no systematic differences. [Done]**

**Response:**

**Original dataset: 30,077**

**After deleting missing values: 16,038**

**Included (kept): 15,386 (95.9%)**

**Excluded (removed): 652 (4.1%)**

**Supplementary Table 1. Distribution of included vs. excluded participants across study variables (*p < 0.001; ns = not significant).**

| **Variable** | **Included n (%)** | **Excluded n (%)** | **p-value** | **Significance** |
| --- | --- | --- | --- | --- |
| **Place of Residence** | **10,080 (65.5%)** | **375 (57.5%)** | **<0.001** | ******* |
| **Division** | **2,352 (15.3%)** | **90 (13.8%)** | **<0.001** | ******* |
| **Highest Educational Level** | **7,004 (45.5%)** | **369 (56.6%)** | **<0.001** | ******* |
| **Religion** | **13,727 (89.2%)** | **629 (96.5%)** | **<0.001** | ******* |
| **Literacy** | **9,541 (62.0%)** | **544 (83.4%)** | **<0.001** | ******* |
| **Wealth Index** | **3,262 (21.2%)** | **247 (37.9%)** | **<0.001** | ******* |
| **Husband Desire for Children** | **13,409 (87.2%)** | **648 (99.4%)** | **<0.001** | ******* |
| **Exposure** | **11,167 (72.6%)** | **600 (92.0%)** | **<0.001** | ******* |
| **Husband Education Level** | **4,855 (31.6%)** | **229 (35.1%)** | **<0.001** | ******* |
| **Respondent Currently Working** | **10,515 (68.3%)** | **506 (77.6%)** | **<0.001** | ******* |
| **Respondent Current Age** | **3,379 (22.0%)** | **186 (28.5%)** | **<0.001** | ******* |
| **Husband Age** | **8,566 (55.7%)** | **429 (65.8%)** | **<0.001** | ******* |
| **Number of Children** | **11,324 (73.6%)** | **589 (90.3%)** | **<0.001** | ******* |
| **Occupation of Husband** | **6,560 (42.6%)** | **254 (39.0%)** | **<0.001** | ******* |
| **Contraceptive Method** | **8,443 (54.9%)** | **536 (82.2%)** | **<0.001** | ******* |
| **Early Marriage** | **12,051 (78.3%)** | **500 (76.7%)** | **0.345** | **ns** |
| **Risk of Pregnancy (Target)** | **4,099 (26.6%)** | **102 (15.6%)** | **<0.001** | ******* |

Thank you for this important suggestion. We have added a comparison table (**Supplementary Table 1**) between the excluded (n=652, 4.1%) and included (n=15,386, 95.9%) groups. As shown in Table X, while 16 of 17 variables showed statistically significant differences (p<0.001), this is a well-documented pattern in DHS survey data known as Missing Not At Random (MNAR), where more educated and urban respondents tend to provide more complete responses to sensitive questions. Three key points justify proceeding with the analysis:

1. The excluded group is SMALL - only 4.1% of the total sample (652 of 16,038 records).

2. The most important predictor - Early Marriage - showed NO significant difference between groups (p=0.345), confirming the primary study variable is unaffected by exclusion.

3. The direction of difference is consistent with known DHS survey response patterns and does not introduce directional bias in the outcome.

We have acknowledged this limitation in the manuscript.

**3) With data loss of 50%, the claim of 'national representativeness' is threatened. I strongly recommend authors to consider Multiple Imputation techniques instead of list-based deletion. [Done]**

**Response:** Thank you for this important and insightful comment. We acknowledge that the exclusion of observations due to missing data may affect the representativeness of the sample.

The initial dataset consisted of 30,077 observations. Several key predictor variables identified from the literature contained substantial missing values. As the primary objective of this study was to develop and evaluate machine learning models using complete and consistent input data, we employed a complete-case analysis approach and excluded observations with missing values. This approach is commonly used in machine learning studies to avoid complications arising from imputation-induced bias and inconsistencies in feature distributions.

After removing cases with missing data, the sample size was reduced to 16,038. Subsequently, 652 duplicate records were removed, resulting in a final analytical sample of 15,386 observations.

To assess potential selection bias, we conducted a comparison between included and excluded observations based on key sociodemographic variables (**Supplementary Table 1**). The results indicate no substantial differences between the two groups, suggesting that the impact on representativeness is limited.

We agree that multiple imputation is a robust alternative for handling missing data. However, given the study’s focus on predictive modeling and the complexity of integrating imputation within cross-validation pipelines, it was not implemented in the current analysis. This limitation has now been acknowledged in the revised manuscript, and future research may explore multiple imputation approaches to enhance robustness.

**4) Please clarify the distribution of the target variable (Early Birth) in the final sample of 8,000. If the classes are imbalanced, what specific ML techniques are used to address this? [Done]**

**Response:**

We appreciate the comment. The target variable for this study was revised from *Early Birth* to *Risk of Pregnancy*. The distribution of the outcome variable was carefully assessed prior to model development to evaluate potential class imbalance. In the full analytical sample (n = 15,386), 11,287 observations (73.4%) belonged to Class 0 (low-risk), while 4,099 observations (26.6%) were in Class 1 (high-risk pregnancy), indicating a moderate class imbalance.

To ensure robust model evaluation, the dataset was split into training (80%) and testing (20%) subsets using stratified sampling, preserving the original class proportions. The training set (n = 12,308) included 73.4% Class 0 and 26.6% Class 1, and the test set (n = 3,078) mirrored this distribution.

To address the observed imbalance in the training data, the **Synthetic Minority Over-sampling Technique (SMOTE)** was applied. A sampling strategy of 0.75 was used, increasing minority class instances from 3,279 to 6,771, resulting in a more balanced training set (Class 0: 57.1%, Class 1: 42.9%; total n = 15,800). The test set remained unaltered to ensure an unbiased evaluation of model performance.

This resampling approach allowed the models to better learn patterns associated with the minority class while minimizing overfitting and maintaining external validity. The flow of data preprocessing, splitting, and resampling is summarized in **Fig 2**.

**Fig 2 : Data Distribution of target variable (risk of birth at early pregnancy)**

**1) Hope the author explains the order of applying SMOTE (before/after split). [Done]**

**Response:** As, any data preprocessing that learns from data (SMOTE, scaling, PCA, feature selection) must be applied only on training data, never before splitting because that cause data leakage. For this reason, I applied SMOTE in only training dataset, which is clearly shown in study Framework **(Fig 1)**

**2) How the author consolidates the results of the 3 methods (Lasso, Chi-Square, dan Boruta) [Done].**

**Response:** We appreciate the comment and the valuable suggestion. The primary objective of this study was to compare the performance of ensemble and non-ensemble machine learning models across three distinct feature selection methods: Lasso (F1), Chi-Square (F2), and Boruta (F3). Consequently, the model performance for each feature set was presented separately to maintain methodological clarity and ensure a fair comparison. Consolidating the results across methods would obscure the relationship between feature selection technique and model performance, which is central to our study.

We acknowledge that presenting results separately may reduce readability. To address this, we have revised the Results section to minimize repetition and provide a more synthesized summary. Specifically, we now clearly highlight that **CART demonstrated the best performance with Lasso-selected features**, whereas **GBM consistently performed best with both Chi-Square and Boruta feature sets**. This revision preserves the comparative objective of the study while improving clarity and interpretability for readers.

• **Evaluation Metrics for Imbalance: Given the imbalance in 'early birth' cases, I recommend including the Precision-Recall Curve (PRC) and AUPRC in addition to AUC-ROC to provide a more rigorous assessment of the model’s predictive power for the minority class. [Done]**

**Response:** We appreciate the suggestion regarding evaluation metrics for imbalanced outcomes. In response, we have added Precision-Recall Curves (PRC) and corresponding Area Under the PRC (AUPRC) values to complement the AUC-ROC metrics, providing a more rigorous assessment of model performance for the minority class. These results are now included in the Results section, both in tabular form and as graphical representations in Figures 8, 9, and 10.

Results
• Methodological Clarity:
1) Table 3, it is not clear whether the final features used for the ML model are an intersection or a combination of the three selection methods. Please explicitly state the final strategy for feature integration. [Done]

**Response:** We appreciate the comment. In this study, three distinct feature selection techniques—**Lasso, Chi-Square, and Boruta**—were applied separately to the training dataset. Rather than taking the intersection or common features across these methods, we **created three separate feature groups**, one from each technique. Machine learning models were then trained and evaluated on each feature group independently.

This approach allowed a direct comparison to determine **which models perform best with which feature set**. Specifically, **CART demonstrated the best performance with Lasso-selected features**, whereas **GBM consistently performed best with both Chi-Square and Boruta feature sets**. By evaluating models separately for each feature selection technique, we were able to preserve clarity and provide actionable insights into the optimal combination of feature selection and modeling approach.

2) Lasso Interpretation: The author mentions the use of **P-Value and Confidence Interval** for the Lasso technique. Since Lasso typically performs shrinkage compared to traditional hypothesis testing, please explain the statistical method used to obtain this P-value. **[Done]**

**Response:** We appreciate the comment. To clarify, **no p-values or confidence intervals were calculated for the Lasso feature selection**, as Lasso is a shrinkage-based method that assigns zero or near-zero coefficients to less important features. The Lasso model was applied to all 16 candidate features using **10-fold cross-validation** to determine the **optimal alpha value (α = 0.001831)** and a **leakage threshold of 0.85**. Feature importance scores indicate the relative influence of each variable on the target outcome.

From the **Fig 3**, **12 features were retained** (green bars), while three features—Religion, Division, and Types of Places of Residence—had coefficients reduced to zero (gray bars) and were therefore excluded. One feature (Respondent Current Age) exceeded the leakage threshold (red bar) and was removed to prevent data leakage.

The retained features reflect those most predictive of the outcome, while less influential or potentially “leaking” variables were systematically excluded. No p-values or confidence intervals are associated with Lasso coefficients, as the method relies on **shrinkage** rather than traditional hypothesis testing.

**3) Boruta Specifics: Regarding the Boruta algorithm, please specify the status of the selected features (for example, are only 'Confirmed' features included, or are 'Tentative' features also considered?) [Done]**

**Response:** We appreciate the comment. In this study, the **Boruta algorithm** was applied for feature selection, and **only the “Confirmed” features** were retained for model development and shown in the feature importance plots. **Tentative features were not included**, ensuring that only the most robust predictors were used for training the models.

**4) Data Consistency: Please ensure that all variables highlighted as significant in Figures 2 and 3 are reported consistently in Table 3. Any differences between the visualization and the final feature set must be justified. [Done]**

**Response:** We have carefully ensured consistency between the figures and **Table 3.** Features from **Fig 3** (Lasso) and **Fig 4** (Boruta) with coefficient values approaching zero were removed and are therefore not included in Table 3 or in the analysis.

**Discussion**

**• Please strengthen the discussion by comparing findings in other countries and explaining the arguments**

**Response:** We have strengthened the Discussion section by incorporating comparisons with findings from other countries, particularly from South Asia and sub-Saharan Africa. We also expanded the explanation of these findings by highlighting contextual factors such as socio-cultural norms, gender roles, and access to information and healthcare services. These revisions improve the depth of interpretation and enhance the relevance of our results in a broader global context.

**Conclusion**

**In the conclusion it is explained that the AUC of 0.95 for one CART model is very high for complex survey data. The authors should discuss whether this performance may be due to overfitting or whether specific hyperparameter tuning has been performed. Next, please compare this with the performance of ensemble models that typically outperform CART.**

**Response:** Thank you for this important observation. We acknowledge that an AUC value as high as 0.95 for a CART model may raise concerns regarding potential overfitting, particularly in the context of complex survey data. In the revised manuscript, we have addressed this issue by incorporating cross-validation results (Table 4) alongside independent prediction performance (Table 5) to ensure model robustness and generalizability.

Specifically, the use of cross-validation helps mitigate overfitting by evaluating model performance across multiple data splits rather than relying on a single training set. Furthermore, the prediction results demonstrate more realistic and stable performance estimates. We also note that, consistent with expectations, ensemble models (e.g., Random Forest and GBM) generally outperform the single CART model in terms of overall predictive performance, particularly in AUC and AUPRC, due to their ability to capture complex patterns and reduce variance.

These additions strengthen the reliability of our findings and provide a more balanced comparison between CART and ensemble approaches.

**• Please add your suggestions for Bangladesh government**

**Response:** Policy relevant implications are delaying age at first birth through enforced minimum marriage ages, expanding female education, and improving facility based delivery in high risk districts could substantially curb early childbirth. Targeted cash transfer schemes for the poorest households may also mitigate the wealth gradient. Collectively, these interventions align with the Sustainable Development Goal 3 target of reducing adolescent birth rates and improving maternal child health outcomes.

Reviewer’s 2 Response

Reviewer #2: Several feature selection methods are presented in the manuscript; however, the rationale for selecting these specific feature selection models is not adequately justified. The authors are encouraged to clearly explain the criteria and reasoning behind the choice of these models and their relevance to the study objectives. [Done]

**Response:**

Most of variables are categorial so use Chi-square feature selection technique to find out most relevant dependent features. Lasso feature selection technique works like as shrinkage algorithm,

**Lasso regression was applied for feature selection due to its ability to reduce model complexity and prevent overfitting through L1 regularization. A key advantage of Lasso regression is its capacity to automatically identify the most influential predictors, thereby improving model interpretability and stability** [1]. LASSO regression has been widely used in preterm birth and public health prediction models for simultaneous coefficient shrinkage and feature selection [2]

The chi‑square test is a filter‑based feature selection method for categorical predictors. It evaluates the bivariate association between each predictor and the outcome (early birth vs term) and keeps variables with statistically significant dependence. In public health, this is very common as a first‑stage screening step to remove clearly irrelevant variables before building multivariable models or machine‑learning pipelines.[3][4]

Bivariate chi‑square tests are routinely used in maternal and child health studies to screen variables associated with preterm birth before multivariable modeling.” For example:

- A preterm birth study that analyzed factors such as parity, pregnancy spacing, and infection using chi‑square tests, then proceeded to logistic regression for effect estimation

Boruta is a **wrapper feature selection method built around Random Forest**. It compares each original variable’s importance to “shadow” (randomly permuted) variables and keeps only those that are significantly more important. This is useful for early birth prediction because:

- It can handle **non‑linear relationships and interactions** that traditional regression may miss.
- It is **conservative**, aiming to identify all relevant variables which aligns with the epidemiologic goal of not missing important risk factors.

Boruta has been used in public‑health prediction models to identify important socio‑demographic and behavioral factors from survey‑based data.” For example:

- A health‑services study on pregnant women where Boruta was used as the main feature selection method to identify key predictors of health‑service access (wealth, media exposure, region, education, alcohol use).[5]
- A machine‑learning analysis of low-birth-weight prediction that used Boruta on DHS‑like data to select eleven significant maternal and child features (age, education, wealth index, age at first birth, BMI, etc.), and then trained prediction models on this reduced set.

These are directly in the maternal/child health domain and closely analogous to early birth prediction. Using complementary filter, embedded, and wrapper feature‑selection strategies is consistent with recent machine‑learning work in maternal and public health prediction, where LASSO, tree‑based importance, and Boruta have all been successfully applied to identify key risk factors for preterm birth, low birth weight, and health‑service use.

[1]	X. Teng, M. Liu, Z. Wang, and X. Dong, “Machine learning prediction of preterm birth in women under 35 using routine biomarkers in a retrospective cohort study,” *Sci. Rep.*, vol. 15, no. 1, p. 10213, Mar. 2025, doi: 10.1038/s41598-025-92814-y.

[2]	Y. Feng *et al.*, “Machine learning-based prediction of preterm birth risk using methylation changes in neonatal cord blood CpG sites,” *BMC Pregnancy Childbirth*, vol. 25, no. 1, p. 784, Jul. 2025, doi: 10.1186/s12884-025-07884-7.

[3]	P. Sethi and M. Jain, “A Comparative Feature Selection Approach for the Prediction of Healthcare Coverage,” in *Information Systems, Technology and Management*, vol. 54, S. K. Prasad, H. M. Vin, S. Sahni, M. P. Jaiswal, and B. Thipakorn, Eds., in Communications in Computer and Information Science, vol. 54. , Berlin, Heidelberg: Springer Berlin Heidelberg, 2010, pp. 392–403. doi: 10.1007/978-3-642-12035-0_41.

[4]	R. Spencer, F. Thabtah, N. Abdelhamid, and M. Thompson, “Exploring feature selection and classification methods for predicting heart disease,” *Digit. Health*, vol. 6, p. 2055207620914777, Jan. 2020, doi: 10.1177/2055207620914777.

[5]	A. K. Kassaw *et al.*, “Optimizing machine learning models for predicting health service access and determinants among pregnant women in rural Ethiopia,” *Sci. Rep.*, vol. 15, no. 1, p. 40559, Nov. 2025, doi: 10.1038/s41598-025-24245-8.

[6]	E. A. Taye *et al.*, “Application of the random forest algorithm to predict skilled birth attendance and identify determinants among reproductive-age women in 27 Sub-Saharan African countries; machine learning analysis,” *BMC Public Health*, vol. 25, no. 1, p. 901, Mar. 2025, doi: 10.1186/s12889-025-22007-9.

[7]	S. Naznin, M. J. Uddin, I. Ahmad, and A. Kabir, “Analyzing and forecasting under-5 mortality trends in Bangladesh using machine learning techniques,” *PLOS ONE*, vol. 20, no. 2, p. e0317715, Feb. 2025, doi: 10.1371/journal.pone.0317715.

[8]	A. O. Khadidos, F. Saleem, S. Selvarajan, Z. Ullah, and A. O. Khadidos, “Ensemble machine learning framework for predicting maternal health risk during pregnancy,” *Sci. Rep.*, vol. 14, no. 1, p. 21483, Sep. 2024, doi: 10.1038/s41598-024-71934-x.

[9]	X. Zhou *et al.*, “Support vector machine deep mining of electronic medical records to predict the prognosis of severe acute myocardial infarction,” *Front. Physiol.*, vol. 13, p. 991990, Sep. 2022, doi: 10.3389/fphys.2022.991990.

**2. The implementation of the machine learning model lacks sufficient technical detail. It is recommended that this section be rewritten with greater technical depth and clarity, including a more systematic explanation of the modeling process. [Done]**

**Response:** Yes, the methodology section has been revised to include detailed descriptions of the machine learning models. The modeling process is now presented systematically, providing greater technical depth and clarity.

**3. Furthermore, the discussion and conclusion sections do not adequately reflect the study objectives. The authors should reconsider revising these sections to explicitly link the key findings to the original objectives and to highlight the practical and scientific implications of the study. [Done]**

**Response:** We have revised both the Discussion and Conclusion sections to explicitly link the key findings to the original study objectives. The revisions highlight how the identified determinants address the aim of predicting early childbirth using BDHS 2022 data and emphasize the practical and scientific implications, including the utility of machine learning models for informing targeted interventions and policy decisions.

Reviewer’s 3 Response

**Reviewer #3:**

1. The authors do not describe the study in a manner that is accessible or easy to understand. It appears to be a cross-sectional or observational study; this should be stated explicitly and it affects the reader's ability to interpret and understand what is a critically important area. [Done]

**Answer:**

**Research Design**

This study employed a cross-sectional design using nationally representative survey data to examine the determinants and predict pregnancy risk among women. Pregnancy risk was defined based on maternal age at childbirth, where women aged ≤18 years or ≥40 years were considered high-risk, and those aged 19–39 years were considered low-risk. The analysis included socio-demographic and health-related factors, and statistical as well as machine learning methods were applied to identify key predictors and estimate the likelihood of high-risk pregnancy.

**Study Framework**

The overall analytical framework of the study is presented in Figure 1. The analysis began with the BDHS 2022 dataset, consisting of 30,078 observations. Pregnancy risk was constructed using the respondent’s current age from the IR dataset. During data preprocessing, 14,040 observations with missing values and 652 duplicate records were removed, resulting in a final dataset of 15,386 observations.

The processed data were then split into training (80%; n = 12,308) and testing (20%; n = 3,078) sets, where the testing set was sealed until the final evaluation stage. Feature selection was conducted on the training data using LASSO (12 features), Chi-square (13 features), and Boruta (10 features) methods.

To ensure model robustness, a 10-fold stratified cross-validation approach was applied. In each iteration, 9 folds were used for training with SMOTE and feature scaling, while 1 fold was used for validation without SMOTE. This process was repeated 10 times, and average performance metrics along with standard deviation were computed.

Subsequently, multiple machine learning models were trained, including non-ensemble methods (CART, SVM, KNN, and Naïve Bayes) and ensemble methods (Random Forest, GBM, AdaBoost, and XGB). Model predictions were evaluated on the testing set using accuracy, precision, recall, F1-score, AUROC, and AUPRC. Finally, statistical validation was performed using McNemar’s test, confusion matrix analysis, ROC curves, and precision–recall curves to compare model performance.

2. The rationale for using machine learning is not fully developed and it appears to have supplanted a traditional epidemiological approach such as traditional logistic regression without explaining the added value. [Done]

**Answer:** Thank you for this insightful comment. We agree that traditional logistic regression is a well-established method in epidemiological research for examining associations between predictors and outcomes. However, the primary objective of this study is predictive modeling rather than causal inference. Logistic regression requires assumptions such as linearity in the log-odds and pre-specification of interaction terms, which may limit its ability to capture complex nonlinear relationships among socio-demographic and behavioral variables. In contrast, machine learning methods can automatically model nonlinear patterns and high-order interactions without strong parametric assumptions, potentially improving predictive accuracy. To strengthen the methodological justification, we have revised the manuscript to clarify the predictive focus of the study and the complementary role of machine learning approaches in enhancing model performance beyond traditional regression techniques.

**3. It is not clear how variables were coded, how continuous variables were categorised or whether survey weights were applied. [Done]**

**Response:** The coding of variables, categorization of continuous variables, and application of survey weights are now clearly described in **Table 1** of the revised manuscript.

**4. the SMOTE use requires to be justified and explained more clearly. Was it applied before or after cross-validation? [Done]**

**Response:** As, any data preprocessing that learns from data (SMOTE, scaling, PCA, feature selection) must be applied only on training data, never before splitting because that cause data leakage. For this reason, I applied SMOTE in only training dataset, which is clearly shown in study Framework **(Fig 1)**

5. There was no discussion of sample size or justification as to whether the final sample was likely to be sufficient for purposes. [Done]

**Response:**

We appreciate the comment. The target variable for this study was revised from *Early Birth* to *Risk of Pregnancy*. The distribution of the outcome variable was carefully assessed prior to model development to evaluate potential class imbalance. In the full analytical sample (n = 15,386), 11,287 observations (73.4%) belonged to Class 0 (low-risk), while 4,099 observations (26.6%) were in Class 1 (high-risk pregnancy), indicating a moderate class imbalance.

To ensure robust model evaluation, the dataset was split into training (80%) and testing (20%) subsets using stratified sampling, preserving the original class proportions. The training set (n = 12,308) included 73.4% Class 0 and 26.6% Class 1, and the test set (n = 3,078) mirrored this distribution.

To address the observed imbalance in the training data, the **Synthetic Minority Over-sampling Technique (SMOTE)** was applied. A sampling strategy of 0.75 was used, increasing minority class instances from 3,279 to 6,771, resulting in a more balanced training set (Class 0: 57.1%, Class 1: 42.9%; total n = 15,800). The test set remained unaltered to ensure an unbiased evaluation of model performance.

This resampling approach allowed the models to better learn patterns associated with the minority class while minimizing overfitting and maintaining external validity. The flow of data preprocessing, splitting, and resampling is summarized in **Fig 2**.

6. Confidence intervals were not included for descriptive statistics. There was no explicit discussion of confounding or bias and how these were overcome. The results relay on tabular display without any meaningful insight e.g. why there may be different prevalences in some geographical areas over others. This alienates readers who are not familiar with the locale and geography, which would be the majority of the international readership.

**Response :**

**1. Inclusion of Confidence Intervals (Descriptive Statistics)**

We have now incorporated **95% confidence intervals (CIs)** for all descriptive prevalence estimates in the revised manuscript **(Supplementary Table 1 & Supplementary Table 2).** This includes:

- Overall prevalence of pregnancy risk
- Division-wise prevalence
- Socio-demographic characteristics (education, residence, wealth)

These additions improve the statistical rigor and allow readers to assess the **precision and variability** of estimates.

**2. Addressing Confounding (Analytical Approach)**

To explicitly account for confounding, we applied a **multivariable logistic regression model**, where all relevant predictors were included simultaneously.

- The results are presented as Adjusted Odds Ratios (AORs) **(Supplementary Table 3)**
- This approach ensures that the effect of each variable is interpreted **while controlling for other covariates**

We have now clarified this in the **Methods section (Statistical Analysis subsection)**.

**3. Discussion of Potential Bias**

We have added a dedicated paragraph in the **Discussion section** addressing potential sources of bias:

- **Selection bias**: Due to complete-case analysis after removal of missing values
- **Information bias**: Possible recall bias in self-reported survey data
- **Residual confounding**: Some unmeasured variables (e.g., cultural practices) may still influence results

We also clarified that these limitations are consistent with large-scale survey-based studies such as BDHS.

**4. Improved Interpretation of Geographic Variation**

To enhance interpretability for an international audience, we expanded the discussion explaining **why prevalence varies across divisions**:

- Higher risk in regions such as Rangpur and Mymensingh may be linked to:
  - Lower educational attainment
  - Higher prevalence of early marriage
  - Limited access to healthcare services
- Lower prevalence in Dhaka may reflect:
  - Better healthcare infrastructure
  - Higher literacy and socioeconomic status

These contextual explanations are now included in the **Results and Discussion sections**.

**5. Improved Presentation (Beyond Tables)**

To avoid over-reliance on tabular data, we have:

- Added a **figure (****Supplementary Fig 1)** illustrating division-wise prevalence with 95% CIs
- Included clear narrative interpretation alongside tables and figures
- Highlighted key findings rather than only presenting numerical results

This improves accessibility for readers unfamiliar with the geographic and social context of Bangladesh.

7. There is an overwhelming emphasis on machine learning (to the detriment of more traditional epidemiological approaches, as detailed above), but these are not portrayed in a way that is accessible to the non-technical reader. I would wish to know the values of different models, whether results between models are meaningful, whether the preferred model can be used in a practical setting and how the modelling could be used by policy makers. I think the latter would add a really powerful punch to this study. As I mentioned earlier, this is a critically important area for research and comment and I am very excited to see new technologies being brought into play here.

**Response:** Thank you for this constructive and encouraging comment. We agree that the presentation of machine learning (ML) results should be more accessible and better integrated with practical and policy relevance. We have substantially revised the manuscript to address these concerns.

**1. Improving Accessibility for Non-Technical Readers**

We have simplified the presentation of ML methods in the revised manuscript by:

- Providing a brief, intuitive explanation of each model (e.g., decision trees, support vector machines)
- Focusing on interpretation of results rather than algorithmic complexity
- Highlighting key findings in plain language within the Results section

This ensures that readers without a technical background can understand the relevance and implications of the models.

**2. Comparison and Value of Different Models**

We compared multiple models (e.g., CART, SVM, KNN, Naïve Bayes) using standard performance metrics including **accuracy, precision, recall, F1-score, and AUC**.

To assess whether differences in model performance were statistically meaningful, we applied the **McNemar test (S5, S6, S7 Table)**, which evaluates whether two models differ significantly in their classification errors on the same dataset.

The results showed that:

- Some models demonstrated **comparable performance**, indicating robustness of findings
- Where differences existed, they were tested for **statistical significance rather than relying solely on numerical differences**

This addition strengthens the validity of model selection.

**3. Selection of the Preferred Model**

The final model was selected based on:

Overall predictive performance (especially F1-score and AUC) mentioned in Result section (subsection **Recommended Machine Learning Model**)

- Stability across cross-validation
- Statistical comparison using the McNemar test

We have clarified that the chosen model provides a **balance between predictive accuracy and interpretability**, which is essential for real-world application.

**4. Practical Applicability of the Model**

We have expanded the discussion to clarify how the selected model can be used in practice:

- The model can be applied to **identify women at high risk of pregnancy complications** using readily available socio-demographic variables
- It can support **early screening and targeted interventions** at the community or healthcare facility level
- The use of relatively simple predictors enhances feasibility in **low-resource settings** such as Bangladesh

**5. Policy Implications (Strengthened Contribution)**

We have added a dedicated paragraph highlighting how the modeling approach can inform policy:

- Policymakers can use the model to **prioritize high-risk geographic regions** (e.g., Rangpur, Mymensingh)
- The findings emphasize the importance of **female education, delaying early marriage, and improving healthcare access**
- ML-based risk prediction tools can be integrated into **national health information systems** to support data-driven decision-making
