# Supplementary material for: Uncovering the determinants of high-risk age at childbirth in Bangladesh: A machine learning analysis of the BDHS 2022 data
Source: PLoS One. 2026 Aug 20;21(8):e0356362. doi: 10.1371/journal.pone.0356362 (PMC13492789; doi:10.1371/journal.pone.0356362)
Supplement: Supplementary file 12 [file pone.0356362.s023.docx]

Comments/Suggestion Journal Plos One

Uncovering the Determinants of Early Birth in Bangladesh: A Machine Learning Analysis of BDHS 2022 Dataset

Title:

- Ok

Abstract:

- ok

Introduction:

- ok

Methods

- Ok

Results

- Ok

Discussion:

•	Ok

Conclusion

- Ok

Recommendation: Accept
