# Supplementary material for: Uncovering the determinants of high-risk age at childbirth in Bangladesh: A machine learning analysis of the BDHS 2022 data
Source: PLoS One. 2026 Aug 20;21(8):e0356362. doi: 10.1371/journal.pone.0356362 (PMC13492789; doi:10.1371/journal.pone.0356362)
Supplement: Peer review history

# Peer review: Decision Letter 0

**DOI:** 10.1371/journal.pone.0356362.r001

**Authors:** Ammal Metwally

19 Feb 2026

Dear Dr. Siam,

Thank you for submitting your manuscript to PLOS ONE. After careful consideration, we feel that it has merit but does not fully meet PLOS ONE’s publication criteria as it currently stands. Therefore, we invite you to submit a revised version of the manuscript that addresses the points raised during the review process.

plosone@plos.org. When you're ready to submit your revision, log on to https://www.editorialmanager.com/pone/ and select the 'Submissions Needing Revision' folder to locate your manuscript file.

- A letter that responds to each point raised by the academic editor and reviewer(s). You should upload this letter as a separate file labeled 'Response to Reviewers'.
- A marked-up copy of your manuscript that highlights changes made to the original version. You should upload this as a separate file labeled 'Revised Manuscript with Track Changes'.
- An unmarked version of your revised paper without tracked changes. You should upload this as a separate file labeled 'Manuscript'.

If you would like to make changes to your financial disclosure, please include your updated statement in your cover letter. Guidelines for resubmitting your figure files are available below the reviewer comments at the end of this letter.

If applicable, we recommend that you deposit your laboratory protocols in protocols.io to enhance the reproducibility of your results. Protocols.io assigns your protocol its own identifier (DOI) so that it can be cited independently in the future. For instructions see: https://journals.plos.org/plosone/s/submission-guidelines#loc-laboratory-protocols. Additionally, PLOS ONE offers an option for publishing peer-reviewed Lab Protocol articles, which describe protocols hosted on protocols.io. Read more information on sharing protocols at https://plos.org/protocols?utm_medium=editorial-email&utm_source=authorletters&utm_campaign=protocols.

We look forward to receiving your revised manuscript.

Kind regards,

Ammal Mokhtar Metwally, Ph.D (MD)

Academic Editor

PLOS One

Journal Requirements:

When submitting your revision, we need you to address these additional requirements.

1.Please ensure that your manuscript meets PLOS ONE's style requirements, including those for file naming. The PLOS ONE style templates can be found at

https://journals.plos.org/plosone/s/file?id=wjVg/PLOSOne_formatting_sample_main_body.pdf and

https://journals.plos.org/plosone/s/file?id=ba62/PLOSOne_formatting_sample_title_authors_affiliations.pdf

2. Please note that PLOS One has specific guidelines on code sharing for submissions in which author-generated code underpins the findings in the manuscript. In these cases, we expect all author-generated code to be made available without restrictions upon publication of the work. Please review our guidelines at https://journals.plos.org/plosone/s/materials-and-software-sharing#loc-sharing-code and ensure that your code is shared in a way that follows best practice and facilitates reproducibility and reuse.

3. Your ethics statement should only appear in the Methods section of your manuscript. If your ethics statement is written in any section besides the Methods, please move it to the Methods section and delete it from any other section. Please ensure that your ethics statement is included in your manuscript, as the ethics statement entered into the online submission form will not be published alongside your manuscript.

4. We notice that your supplementary figures are uploaded with the file type 'Figure'. Please amend the file type to 'Supporting Information'. Please ensure that each Supporting Information file has a legend listed in the manuscript after the references list.

5. If the reviewer comments include a recommendation to cite specific previously published works, please review and evaluate these publications to determine whether they are relevant and should be cited. There is no requirement to cite these works unless the editor has indicated otherwise.

Additional Editor Comments:

<h2>1. Abstract</h2>

The abstract is clear and includes data source, outcome, methods, and key findings, but the framing can be sharper and more neutral. It should more explicitly specify study design and sample characteristics and avoid promotional wording to align with PLOS ONE’s scientific tone. **It is advisable to r** eplace phrases like “key ingredients” or “exceptionally effective” with neutral alternatives such as “most influential factors” and “showed the highest predictive performance,” and add a phrase such as “cross-sectional secondary analysis of 8,000 ever-married women from BDHS 2022.”

<h2>2. Introduction</h2>

The Introduction is informative but suffers from repetition and does not clearly converge on the specific knowledge gap and novelty. Adverse outcomes and general determinants (education, poverty, rural residence) are reiterated several times, while the unique contribution—using BDHS 2022 with ML model comparison—is underemphasized. **It is advisable to r** estructure the section into four paragraphs (global burden → Bangladesh context → known determinants → explicit gap and objectives) and add a clear gap sentence such as: “No previous study has systematically compared machine learning algorithms to predict early first birth using BDHS 2022 data.”

Some language in the Introduction is informal or imprecise for a scientific article. Informal terms (“first kid”, “big effect”) and vague phrasing reduce the perceived rigor of the manuscript. **It is advisable to u** se more formal phrasing like “first child,” “substantial impact,” and describe psychosocial consequences in precise terms, e.g., lower educational attainment and reduced economic opportunities.

<h2>3. Methods</h2>

Terminology for the main outcome is inconsistent, with references to “early marriage” instead of “early first birth” in several places. This inconsistency strongly suggests copy-paste from another project and can undermine reviewer confidence in the care taken with the analysis. **It is advisable to s** ystematically replace “early marriage” with “early first birth (≤19 years)” and ensure that all descriptions of classes, SMOTE, and the conceptual framework use the correct outcome term.

The derivation of the final analytic sample of 8,000 women is not fully transparent. A clear description of who was excluded and why (never-married, missing age at first birth, missing covariates, etc.) is needed to interpret the generalizability and risk of selection bias. **It is advisable to a** dd a concise sample-flow description (and ideally a simple diagram), such as: “From 16,038 interviewed women aged 15–49, we excluded never-married women, those without recorded age at first birth, and those with missing values in key covariates, yielding 8,000 ever-married women for analysis.”

The ML pipeline is generally described, but model names, SMOTE usage, and software environment are not always clearly specified.  Ambiguous abbreviations (e.g., “RM”), lack of detail on when SMOTE is applied, and missing information on software packages hinder reproducibility. **It is advisable to s** tandardize abbreviations (LR, CART, RF, GBM, XGB, KNN), explicitly state that SMOTE was applied within each training fold of cross-validation, and specify that analyses were conducted in Python with scikit-learn and XGBoost (with version numbers if possible).

<h2>4. Results</h2>

The narrative for the descriptive statistics is overly detailed and repetitive, listing many cell percentages from the table. This level of detail obscures the main patterns and makes the Results section longer and harder to follow. **It is advisable to c** ondense the description of Table 2 to highlight only the strongest gradients (education, wealth, rural vs urban residence, age at marriage, contraceptive use) in a short interpretive paragraph instead of enumerating many individual percentages.

Some narrative statements (e.g., about which divisions have the highest prevalence) do not perfectly align with the percentages in the tables. Any mismatch between text and tables is a technical inaccuracy that reviewers quickly notice and may question. **It is advisable to r** echeck and rewrite those sentences so they conform to the table, for instance: “Rangpur and Khulna show the highest prevalence of early birth, whereas Sylhet and Dhaka have comparatively lower levels,” if that matches the reported data.

Model performance and feature-selection results are described in lengthy and somewhat repetitive paragraphs. Repeating similar performance metrics for multiple models and feature-selection schemes reduces clarity and can overwhelm the reader. **It is advisable to p** rovide a synthesized comparison such as: “Across all feature-selection methods, CART, particularly when combined with Chi-square–selected features, yielded the highest overall performance, with GBM and RF performing similarly but not consistently outperforming CART.”

<h2>5. Discussion and Conclusion</h2>

The Discussion appropriately revisits key findings but sometimes repeats background material instead of focusing on the interpretation of this study’s specific results. Discussion sections should synthesize how the current findings compare with previous work and what they imply, rather than re-explaining general facts about early childbearing. **It is advisable to r** ephrase generic statements as comparative ones: “Our findings confirm and extend previous evidence that rural residence, lower wealth, and lack of formal education are strongly associated with early first birth in Bangladesh and other LMICs.”

The added value of machine learning (especially CART and GBM) is not fully translated into practical or policy implications. Since the study compares ML models, reviewers will expect a clearer explanation of how these models could be used in targeting interventions or creating risk tools. **It is advisable to a** dd a paragraph explaining that CART trees can be converted into simple decision rules or risk scores based on combinations of education, age at marriage, and wealth, which frontline workers could use to identify adolescents at high risk of early first birth.

The Conclusion includes some informal or conversational phrases that are not ideal for a scientific journal. A concise, neutral conclusion is more appropriate for PLOS ONE and reinforces the scientific tone. **It is advisable to r** eplace phrases such as “beyond the numbers” with a more neutral summary: “A small set of socio-demographic factors—particularly education, age at marriage, household wealth, and contraceptive use—largely determines the risk of first birth before age 19, and ML models like CART can help target prevention efforts.”

<h2>6. Cross-cutting Issues (Language, Cover Letter, Data Availability)</h2>

Overall language quality is acceptable but requires systematic polishing for grammar, syntax, and formal tone. Recurrent minor errors and informal wording may negatively influence reviewers’ perception of the manuscript, even if the substantive content is strong. **It is advisable to u** ndertake a focused language edit to correct subject–verb agreement, unify verb tenses, and replace informal expressions with more formal academic language.

The cover letter still contains visible template instructions rather than polished text. Leaving editorial prompts in the cover letter appears unprofessional and may raise concerns with editors at first glance. **It is advisable to r** ewrite that section into a concise, original paragraph summarizing the study’s aim, novelty (BDHS 2022 + ML comparison), and suitability for PLOS ONE, and remove any placeholder phrases like “briefly describe the main focus.”

There is inconsistency between the data-availability statement in the submission system and in the manuscript text. PLOS ONE requires a single, clear, and accurate description of how data can be accessed; inconsistencies can delay the editorial process. **Example:**  Use one standard DHS formulation everywhere, e.g.: “The data underlying this article are available from The DHS Program (https://dhsprogram.com/data/) upon registration and approval of a brief research proposal.”

[Note: HTML markup is below. Please do not edit.]

Reviewer's Responses to Questions

**Comments to the Author**

1. Is the manuscript technically sound, and do the data support the conclusions?

Reviewer #1: No

Reviewer #2: Partly

Reviewer #3: Partly

**********

2. Has the statistical analysis been performed appropriately and rigorously?

Reviewer #1: No

Reviewer #2: I Don't Know

Reviewer #3: No

**********

3. Have the authors made all data underlying the findings in their manuscript fully available?

The PLOS Data policy

Reviewer #1: Yes

Reviewer #2: Yes

Reviewer #3: Yes

**********

4. Is the manuscript presented in an intelligible fashion and written in standard English?

Reviewer #1: Yes

Reviewer #2: No

Reviewer #3: No

**********

Reviewer #1: Title:

• Ok

Abstract:

• Please explain the research design, sample size, inclusion and exclusion criteria

Introduction:

• At the end of the paragraph in the introduction, please add the research objective

Methods

• Explain the research design

• Important Concerns on Data Pre-processing:

1) "The authors excluded approximately 50% of the initial sample (from 16,038 to 8,000) due to missing information. This massive reduction raises significant concerns regarding Selection Bias.

2) Authors should provide a comparison table between the excluded and included groups to show that there are no systematic differences.

3) With data loss of 50%, the claim of 'national representativeness' is threatened. I strongly recommend authors to consider Multiple Imputation techniques instead of list-based deletion.

4) Please clarify the distribution of the target variable (Early Birth) in the final sample of 8,000. If the classes are imbalanced, what specific ML techniques are used to address this?”

• The methodological framework is comprehensive; however, the authors should clarify important steps in their process:

1) Hope the author explains the order of applying SMOTE (before/after split).

2) How the author consolidates the results of the 3 methods (Lasso, Chi-Square, dan Boruta)

3) The comparison between linear (Logistic) and non-linear (XGBoost) models is correct.

• Evaluation Metrics for Imbalance: Given the imbalance in 'early birth' cases, I recommend including the Precision-Recall Curve (PRC) and AUPRC in addition to AUC-ROC to provide a more rigorous assessment of the model’s predictive power for the minority class.

Results

• Methodological Clarity:

1) n Table 3, it is not clear whether the final features used for the ML model are an intersection or a combination of the three selection methods. Please explicitly state the final strategy for feature integration.

2) Lasso Interpretation: The author mentions the use of P-Value and Confidence Interval for the Lasso technique. Since Lasso typically performs shrinkage compared to traditional hypothesis testing, please explain the statistical method used to obtain this P-value.

3) Boruta Specifics: Regarding the Boruta algorithm, please specify the status of the selected features (for example, are only 'Confirmed' features included, or are 'Tentative' features also considered?).

4) Data Consistency: Please ensure that all variables highlighted as significant in Figures 2 and 3 are reported consistently in Table 3. Any differences between the visualization and the final feature set must be justified.

Discuss

• Please strengthen the discussion by comparing findings in other countries and explaining the arguments

Conclusion

• In the conclusion it is explained that the AUC of 0.95 for one CART model is very high for complex survey data. The authors should discuss whether this performance may be due to overfitting or whether specific hyperparameter tuning has been performed. Next, please compare this with the performance of ensemble models that typically outperform CART.

• Please add your suggestions for Bangladesh government

Recommendation: Major revision

This study addresses a critical public health issue in Bangladesh using a machine learning approach on the BDHS 2022 dataset. While the integration of multiple feature selection methods and the use of multilevel k-fold validation are commendable, there are several technical issues related to data preprocessing, potential data leakage, and terminological consistency that must be addressed to ensure the validity of the findings.

Reviewer #2: Several feature selection methods are presented in the manuscript; however, the rationale for selecting these specific feature selection models is not adequately justified. The authors are encouraged to clearly explain the criteria and reasoning behind the choice of these models and their relevance to the study objectives.

The implementation of the machine learning model lacks sufficient technical detail. It is recommended that this section be rewritten with greater technical depth and clarity, including a more systematic explanation of the modeling process.

Furthermore, the discussion and conclusion sections do not adequately reflect the study objectives. The authors should reconsider revising these sections to explicitly link the key findings to the original objectives and to highlight the practical and scientific implications of the study.

Reviewer #3: The authors do not describe the study in a manner that is accessible or easy to understand. It appears to be a cross-sectional or observational study; this should be stated explicitly and it affects the reader's ability to interpret and understand what is a critically important area. The rationale for using machine learning is not fully developed and it appears to have supplanted a traditional epidemiological appraoch such as traditional logistic regression without explaining the added value. It is not clear how variables were coded, how continuous variables were catgorised or whether survey weights were applied. the SMOTE use requires to be justified and explained more clearly. Was it applied before or after cross-validation? There was no discussion of sample size or justification as to whether the final sample was likely to be sufficient for purposes. Confidence intervals were not included for descriptive statistics. There was no explicit discussion of confounding or bias and how these were overcome. The results relay on tabular display without any meaningful insight e.g. why there may be different prevalences in some geographical areas over others. This alienates readers who are not familiar with the locale and geography, which would be the majority of the international readership. There is an overwhelming emphasis on machine learning (to the detriment of more traditional epidemiological appraoches, as detailed above), but these are not portrayed in a way that is accessible to the non-technical reader. I would wish to know the values of diffferent models, whether results between models are meaningful, whether the preferred model can be used in a practical setting and how the modelling could be used by policy makers. I think the latter would add a really powerful punch to this study. As I mentioned earlier, this is a critically important area for research and comment and I am very excited to see new technologies being brought into play here. However, the paper suffers by being written in a manner which is too dense and inaccessible to the reader who is not familiar with this field, and I feel that it is too important to be allowed to be compromised in this way. I believe that this could be an excellent paper and could, if it addresses the points above, be a worthy addition to the evidence base for this incredibly important field. I would encourage the authors to concentrate on addressing some of the statistical areas lacking, but most importantly to write more clearly around the machine learning aspects of their work, to try and help their readers along a journey to acceptance of an important new tool in medical research.

**********

what does this mean?). If published, this will include your full peer review and any attached files.

If you choose “no”, your identity will remain anonymous but your review may still be made public.

**Do you want your identity to be public for this peer review?**  For information about this choice, including consent withdrawal, please see our Privacy Policy

Reviewer #1: No

Reviewer #2: No

Reviewer #3: No

**********

[NOTE: If reviewer comments were submitted as an attachment file, they will be attached to this email and accessible via the submission site. Please log into your account, locate the manuscript record, and check for the action link "View Attachments". If this link does not appear, there are no attachment files.]

To ensure your figures meet our technical requirements, please review our figure guidelines: https://journals.plos.org/plosone/s/figures

You may also use PLOS’s free figure tool, NAAS, to help you prepare publication quality figures: https://journals.plos.org/plosone/s/figures#loc-tools-for-figure-preparation.

NAAS will assess whether your figures meet our technical requirements by comparing each figure against our figure specifications.

# Author comment: Author response to Decision Letter 1

**DOI:** 10.1371/journal.pone.0356362.r002

23 Apr 2026

Editor's Response :

1. Abstract

The abstract is clear and includes data source, outcome, methods, and key findings, but the framing can be sharper and more neutral. It should more explicitly specify study design and sample characteristics and avoid promotional wording to align with PLOS ONE’s scientific tone. It is advisable to replace phrases like “key ingredients” or “exceptionally effective” with neutral alternatives such as “most influential factors” and “showed the highest predictive performance,” and add a phrase such as “cross-sectional secondary analysis of 8,000 ever-married women from BDHS 2022.”

Response: Thank you for your constructive feedback. We have revised the Abstract to improve clarity, precision, and adherence to a neutral scientific tone consistent with PLOS ONE guidelines.

Specifically, we now explicitly state the study design and sample characteristics, describing the analysis as a cross-sectional secondary analysis of BDHS 2022 data and clearly reporting the study population. In addition, we have replaced informal or promotional wording (e.g., “key ingredients,” “exceptionally effective”) with more neutral scientific expressions. These revisions enhance the clarity, transparency, and academic rigor of the abstract.

2. Introduction

The Introduction is informative but suffers from repetition and does not clearly converge on the specific knowledge gap and novelty. Adverse outcomes and general determinants (education, poverty, rural residence) are reiterated several times, while the unique contribution—using BDHS 2022 with ML model comparison—is underemphasized. It is advisable to restructure the section into four paragraphs (global burden → Bangladesh context → known determinants → explicit gap and objectives) and add a clear gap sentence such as: “No previous study has systematically compared machine learning algorithms to predict early first birth using BDHS 2022 data.”

Some language in the Introduction is informal or imprecise for a scientific article. Informal terms (“first kid”, “big effect”) and vague phrasing reduce the perceived rigor of the manuscript. It is advisable to use more formal phrasing like “first child,” “substantial impact,” and describe psychosocial consequences in precise terms, e.g., lower educational attainment and reduced economic opportunities.

Response: Thank you for your valuable comments on our manuscript. We have carefully revised the Introduction to address all concerns.

Specifically, we restructured it into four clear paragraphs (global context, Bangladesh context, determinants, and research gap), removed repetition, and improved the academic tone. We also explicitly clarified the study’s novelty by adding the gap statement: “No previous study has systematically compared machine learning algorithms to predict early first birth using BDHS 2022 data.”

We believe these revisions have improved the clarity and contribution of the manuscript. Thank you for your consideration.

3. Methods

1. Terminology for the main outcome is inconsistent, with references to “early marriage” instead of “early first birth” in several places. This inconsistency strongly suggests copy-paste from another project and can undermine reviewer confidence in the care taken with the analysis. It is advisable to systematically replace “early marriage” with “early first birth (≤19 years)” and ensure that all descriptions of classes, SMOTE, and the conceptual framework use the correct outcome term. [Done]

Response: Thank you for highlighting this important issue. We sincerely apologize for the inconsistency in terminology, which was unintentional and has now been carefully corrected throughout the manuscript.

In the revised version, we have updated and clarified the definition of the outcome variable. Specifically, the target variable has been redefined as “risk at birth”, constructed based on the respondent’s age at childbirth. The variable is coded as 1 (high risk) for women who gave birth at ≤18 years or ≥40 years, and 0 (low risk/normal) for those who gave birth between 19 and 39 years.

All instances of incorrect terminology, including “early marriage,” have been systematically replaced to ensure consistency across the manuscript, including in the descriptions of class labels, SMOTE application, and the conceptual framework. This clarification has been explicitly detailed in the Methodology section under the “Target Variable” subsection.

We appreciate the reviewer’s careful observation, which has helped improve the clarity and consistency of our study.

2. The derivation of the final analytic sample of 8,000 women is not fully transparent. A clear description of who was excluded and why (never-married, missing age at first birth, missing covariates, etc.) is needed to interpret the generalizability and risk of selection bias. It is advisable to add a concise sample-flow description (and ideally a simple diagram), such as: “From 16,038 interviewed women aged 15–49, we excluded never-married women, those without recorded age at first birth, and those with missing values in key covariates, yielding 8,000 ever-married women for analysis.” [Done]

Response: We included 16 independent predictors based on the literature review. Several variables contained missing values; however, no imputation techniques were applied, as prior evidence suggests that certain machine learning models may perform sub optimally with imputed data. The approach to handling missing data is described in the methodology section under “Sample Size and Handling of Missing Data.” Additionally, the process used to derive the final analytical sample is illustrated in Fig 2 through a detailed sample flowchart.

3. The ML pipeline is generally described, but model names, SMOTE usage, and software environment are not always clearly specified. Ambiguous abbreviations (e.g., “RM”), lack of detail on when SMOTE is applied, and missing information on software packages hinder reproducibility. It is advisable to standardize abbreviations (LR, CART, RF, GBM, XGB, KNN), explicitly state that SMOTE was applied within each training fold of cross-validation, and specify that analyses were conducted in Python with scikit-learn and XGBoost (with version numbers if possible). [Done]

Response:

Thank you for this valuable suggestion, which has helped us improve the clarity and reproducibility of our methodology.

In the revised manuscript, we have standardized all model abbreviations to commonly accepted forms, including Classification and Regression Trees (CART), Random Forest (RF), Gradient Boosting Machine (GBM), Adaptive Boosting (AdaBoost), Extreme Gradient Boosting (XGB), and K-Nearest Neighbors (KNN), Naïve Bayes (NB). Ambiguous abbreviations such as “RM” have been removed to avoid confusion.

We have also clarified the implementation of the SMOTE technique in the research design section. Specifically, SMOTE was applied only to the training data, ensuring that no information from the test data was used during model training and thereby preventing data leakage. This detail has now been explicitly stated in the Methods section.

Furthermore, we have added a detailed description of the software environment used for the analysis. All models were implemented in Python, using the scikit-learn library for LR, CART, RF, GBM, and KNN, and the XGB library for XGB. The corresponding version numbers have also been included in the revised manuscript to enhance reproducibility.

All experiments were conducted using Python (version 3.11) on the Google Colaboratory (Colab) platform, utilizing an NVIDIA T4 GPU, Intel Xeon 2.20 GHz CPU, and 12 GB RAM. Data preprocessing and manipulation were performed using Pandas (version 2.2.2) and NumPy (version 2.0.2), while visualization was conducted using Matplotlib (version 3.5.2) and Seaborn (version 0.12.2). Machine learning models, including Logistic Regression (LR), Classification and Regression Trees (CART), Random Forest (RF), Gradient Boosting Machine (GBM), Extreme Gradient Boosting (XGB), and K-Nearest Neighbors (KNN), were implemented using Scikit-learn (version 1.6.1) and XGB (version 2.0.2), with additional deep learning support from TensorFlow (version 2.12).

Model performance was evaluated using 10-fold cross-validation to ensure robust generalization. To address class imbalance, the Synthetic Minority Oversampling Technique (SMOTE) was applied using the imbalanced-learn package (version 0.14.1). Importantly, SMOTE was applied only to the training data within each cross-validation fold, thereby preventing data leakage and preserving the integrity of the test data.These revisions have been incorporated into the Experimental Settings Results section of the manuscript.

We appreciate the reviewer’s insightful comments, which have significantly strengthened the transparency and reproducibility of our study.

4. Results

1. The narrative for the descriptive statistics is overly detailed and repetitive, listing many cell percentages from the table. This level of detail obscures the main patterns and makes the Results section longer and harder to follow. It is advisable to condense the description of Table 2 to highlight only the strongest gradients (education, wealth, rural vs urban residence, age at marriage, contraceptive use) in a short interpretive paragraph instead of enumerating many individual percentages. [Done]

Response: Thank you for this helpful suggestion. In response, we have revised the descriptive statistics section for Table 2 by removing repetitive reporting of individual cell percentages and condensing the narrative. The revised text now emphasizes the key gradients—education, wealth, rural–urban residence, age at marriage, and contraceptive use—through a concise and interpretive summary. This has improved the clarity and readability of the Results section.

2. Some narrative statements (e.g., about which divisions have the highest prevalence) do not perfectly align with the percentages in the tables. Any mismatch between text and tables is a technical inaccuracy that reviewers quickly notice and may question. It is advisable to recheck and rewrite those sentences so they conform to the table, for instance: “Rangpur and Khulna show the highest prevalence of early birth, whereas Sylhet and Dhaka have comparatively lower levels,” if that matches the reported data. [Done]

Response:

Thank you for highlighting this issue. In the revised manuscript, Table 2 has been updated, and all corresponding narrative statements have been carefully rechecked and aligned with the reported percentages. The descriptions have been rewritten to ensure full consistency between the text and the table, minimizing the possibility of any discrepancies. We believe this revision has addressed the concern effectively.

3. Model performance and feature-selection results are described in lengthy and somewhat repetitive paragraphs. Repeating similar performance metrics for multiple models and feature-selection schemes reduces clarity and can overwhelm the reader. It is advisable to provide a synthesized comparison such as: “Across all feature-selection methods, CART, particularly when combined with Chi-square–selected features, yielded the highest overall performance, with GBM and RF performing similarly but not consistently outperforming CART.” [Done]

Response:

Thank you for this valuable suggestion. The primary objective of this study is to compare the performance of ensemble and non-ensemble models across three distinct feature-selection methods, namely Lasso (F1), Chi-square (F2), and Boruta (F3). As a result, model performance has been reported separately for each feature set to maintain methodological clarity and ensure a fair comparison.

However, we acknowledge that this approach may reduce readability. Accordingly, we have revised the Results section to minimize repetition and include a more synthesized summary of findings. In particular, we now clearly highlight that CART demonstrated the best performance with Lasso-selected features, whereas GBM consistently performed best with both Chi-square and Boruta feature sets. This revision improves clarity while preserving the comparative objective of the study.

5. Discussion and Conclusion

The Discussion appropriately revisits key findings but sometimes repeats background material instead of focusing on the interpretation of this study’s specific results. Discussion sections should synthesize how the current findings compare with previous work and what they imply, rather than re-explaining general facts about early childbearing. It is advisable to rephrase generic statements as comparative ones: “Our findings confirm and extend previous evidence that rural residence, lower wealth, and lack of formal education are strongly associated with early first birth in Bangladesh and other LMICs.” The added value of machine learning (especially CART and GBM) is not fully translated into practical or policy implications. Since the study compares ML models, reviewers will expect a clearer explanation of how these models could be used in targeting interventions or creating risk tools. It is advisable to add a paragraph explaining that CART trees can be converted into simple decision rules or risk scores based on combinations of education, age at marriage, and wealth, which frontline workers could use to identify adolescents at high risk of early first birth. The Conclusion includes some informal or conversational phrases that are not ideal for a scientific journal. A concise, neutral conclusion is more appropriate for PLOS ONE and reinforces the scientific tone. It is advisable to replace phrases such as “beyond the numbers” with a more neutral summary: “A small set of socio-demographic factors— particularly education, age at marriage, household wealth, and contraceptive use—largely determines the risk of first birth before age 19, and ML models like CART can help target prevention efforts.”

Response: Thank you for these valuable suggestions. We have revised both the Discussion and Conclusion sections to improve clarity, focus, and scientific tone.

First, the Discussion has been refined to reduce repetition of general background information and instead emphasize the interpretation of our study-specific findings. We have incorporated more comparative statements to align our results with existing literature, highlighting how our findings confirm and extend previous evidence on key determinants such as education, age at marriage, household wealth, and place of residence in Bangladesh and similar LMIC contexts.

Second, we have strengthened the practical and policy implications of the machine learning models. In particular, we added a paragraph explaining how models such as CART can be translated into simple decision rules or risk stratification tools, which can be applied by frontline health workers to identify adolescents at high risk of early first birth based on key socio-demographic characteristics. We also clarified the added value of ensemble models such as GBM in improving predictive accuracy for population-level targeting.

Finally, the Conclusion section has been revised to remove informal language and adopt a more concise, neutral, and scientific tone, in line with journal expectations. The revised conclusion now clearly summarizes that a limited set of socio-demographic factors—particularly education, age at marriage, household wealth, and contraceptive use—are strong predictors of early first birth, and that machine learning approaches can support more targeted prevention strategies.

These revisions improve the overall coherence, rigor, and applicability of the manuscript.

Reviewer 1 Response:

Abstract:

Please explain the research design, sample size, inclusion and exclusion criteria

Response: Thank you for your suggestion. We have revised the Abstract to clearly describe the research design, sample size, and eligibility criteria. Specifically, we now state that the study is a cross-sectional secondary analysis of BDHS 2022 data, and we report the final sample size included in the analysis. We have also briefly outlined the inclusion criteria (ever-married women within the reproductive age group with complete information on study variables) and the exclusi

Attachment Submitted filename: Response of Reviewers.docx

# Peer review: Decision Letter 1

**DOI:** 10.1371/journal.pone.0356362.r003

**Authors:** Ammal Metwally

10 Jun 2026

Dear Dr. Siam,

Thank you for submitting your manuscript to PLOS ONE. After careful consideration, we feel that it has merit but does not fully meet PLOS ONE’s publication criteria as it currently stands. Therefore, we invite you to submit a revised version of the manuscript that addresses the points raised during the review process.

Please submit your revised manuscript by Jul 25 2026 11:59PM. If you will need more time than this to complete your revisions, please reply to this message or contact the journal office at plosone@plos.org. When you're ready to submit your revision, log on to https://www.editorialmanager.com/pone/ and select the 'Submissions Needing Revision' folder to locate your manuscript file.

- A letter that responds to each point raised by the academic editor and reviewer(s). You should upload this letter as a separate file labeled 'Response to Reviewers'.
- A marked-up copy of your manuscript that highlights changes made to the original version. You should upload this as a separate file labeled 'Revised Manuscript with Track Changes'.
- An unmarked version of your revised paper without tracked changes. You should upload this as a separate file labeled 'Manuscript'.

If you would like to make changes to your financial disclosure, please include your updated statement in your cover letter. Guidelines for resubmitting your figure files are available below the reviewer comments at the end of this letter.

If applicable, we recommend that you deposit your laboratory protocols in protocols.io to enhance the reproducibility of your results. Protocols.io assigns your protocol its own identifier (DOI) so that it can be cited independently in the future. For instructions see: https://journals.plos.org/plosone/s/submission-guidelines#loc-laboratory-protocols. Additionally, PLOS ONE offers an option for publishing peer-reviewed Lab Protocol articles, which describe protocols hosted on protocols.io. Read more information on sharing protocols at https://plos.org/protocols?utm_medium=editorial-email&utm_source=authorletters&utm_campaign=protocols.

As the corresponding author, your ORCID iD is verified in the submission system and will appear in the published article. PLOS supports the use of ORCID, and we encourage all coauthors to register for an ORCID iD and use it as well. Please encourage your coauthors to verify their ORCID iD within the submission system before final acceptance, as unverified ORCID iDs will not appear in the published article. *Only*  the individual author can complete the verification step; PLOS staff *cannot*  verify ORCID iDs on behalf of authors.

We look forward to receiving your revised manuscript.

Kind regards,

Ammal Mokhtar Metwally, Ph.D (MD)

Academic Editor

PLOS One

Journal Requirements:

If the reviewer comments include a recommendation to cite specific previously published works, please review and evaluate these publications to determine whether they are relevant and should be cited. There is no requirement to cite these works unless the editor has indicated otherwise.

Please review your reference list to ensure that it is complete and correct. If you have cited papers that have been retracted, please include the rationale for doing so in the manuscript text, or remove these references and replace them with relevant current references. Any changes to the reference list should be mentioned in the rebuttal letter that accompanies your revised manuscript. If you need to cite a retracted article, indicate the article’s retracted status in the References list and also include a citation and full reference for the retraction notice.

Additional Editor Comments:

Dear Authors,

Thank you for submitting the revised version of your manuscript entitled “Uncovering the Determinants of Early Pregnancy and High-Risk Birth in Bangladesh: A Machine Learning Analysis of BDHS 2022 Dataset.” The revised manuscript has improved substantially in response to the reviewers’ and editorial comments. The study now presents a clearer cross-sectional secondary analysis of BDHS 2022 data, provides a more transparent description of the analytic sample, expands the machine-learning workflow, and adds relevant performance metrics for imbalanced classification.

Key weaknesses requiring minor revision

The most important remaining issue is outcome inconsistency. The manuscript title and narrative emphasize early pregnancy/early childbirth, but the Methods define the outcome as high-risk age at childbirth, coded as high risk for ≤18 years or ≥40 years and low risk for 19–39 years. This changes the scientific meaning of the study and must be harmonized before acceptance.

The sample derivation and missing-data explanation remain vulnerable. Excluding 14,040 observations is substantial, and the response should not overstate representativeness or claim minimal bias if many included/excluded variables differ significantly.

The best-performing model is not consistently reported across the abstract, results, response letter, and tables. This should be corrected by specifying whether the “best” model is based on cross-validation, final test-set performance, AUROC, AUPRC, F1, or accuracy.

[Note: HTML markup is below. Please do not edit.]

Reviewers' comments:

Reviewer's Responses to Questions

**Comments to the Author**

Reviewer #1: All comments have been addressed

Reviewer #2: (No Response)

Reviewer #3: All comments have been addressed

**********

2. Is the manuscript technically sound, and do the data support the conclusions?

Reviewer #1: Yes

Reviewer #2: Partly

Reviewer #3: Yes

**********

3. Has the statistical analysis been performed appropriately and rigorously?

Reviewer #1: Yes

Reviewer #2: I Don't Know

Reviewer #3: Yes

**********

4. Have the authors made all data underlying the findings in their manuscript fully available?

The PLOS Data policy

Reviewer #1: Yes

Reviewer #2: Yes

Reviewer #3: Yes

**********

5. Is the manuscript presented in an intelligible fashion and written in standard English?

Reviewer #1: Yes

Reviewer #2: Yes

Reviewer #3: Yes

**********

Reviewer #1: The author has made improvements according to the input I provided very well, including improvements to the abstract, introduction, methods, results, discussion and conclusions. There are no more objections from me.

Reviewer #2: The authors appear to have made a reasonable effort to address the concerns raised by the reviewers. However, several of the revisions seem primarily aimed at providing justifications in response to reviewer comments rather than fully integrating the corresponding changes into the manuscript itself.

Furthermore, the manuscript does not consistently adhere to the formatting requirements and guidelines of PLOS. The authors are encouraged to carefully review the journal's formatting policies and ensure full compliance prior to resubmission.

Reviewer #3: All of the concerns appear to have been adequately addressed by the authors. I am confident that this review meets the standards required by PLOS One.

**********

what does this mean?). If published, this will include your full peer review and any attached files.

If you choose “no”, your identity will remain anonymous but your review may still be made public.

**Do you want your identity to be public for this peer review?**  For information about this choice, including consent withdrawal, please see our Privacy Policy

Reviewer #1: No

Reviewer #2: No

Reviewer #3: **Yes:**  Declan McKeown

**********

[NOTE: If reviewer comments were submitted as an attachment file, they will be attached to this email and accessible via the submission site. Please log into your account, locate the manuscript record, and check for the action link "View Attachments". If this link does not appear, there are no attachment files.]

To ensure your figures meet our technical requirements, please review our figure guidelines: https://journals.plos.org/plosone/s/figures

You may also use PLOS’s free figure tool, NAAS, to help you prepare publication quality figures: https://journals.plos.org/plosone/s/figures#loc-tools-for-figure-preparation.

NAAS will assess whether your figures meet our technical requirements by comparing each figure against our figure specifications.

# Author comment: Author response to Decision Letter 2

**DOI:** 10.1371/journal.pone.0356362.r004

12 Jun 2026

Additional Editor Comments:

1. The most important remaining issue is outcome inconsistency. The manuscript title and narrative emphasize early pregnancy/early childbirth, but the Methods define the outcome as high-risk age at childbirth, coded as high risk for ≤18 years or ≥40 years and low risk for 19–39 years. This changes the scientific meaning of the study and must be harmonized before acceptance.

Response: Thank you for this important observation. We agree that the terminology used in the previous version of the manuscript created inconsistency between the study narrative and the outcome variable definition. The outcome analyzed in this study was maternal age at childbirth, categorized as high-risk (≤18 years or ≥40 years) and low-risk (19–39 years). Therefore, the term “high-risk age at childbirth” more accurately reflects the outcome investigated than “early pregnancy” or “early childbirth.”

To address this concern, we have thoroughly revised the manuscript to harmonize the terminology throughout all sections. Specifically, references to “early pregnancy” and “early childbirth” have been replaced with “high-risk age at childbirth” in the title, abstract, introduction, results, discussion, tables, and supplementary materials where appropriate. We have also clarified the operational definition of the outcome variable in the Methods section to ensure consistency between the study objective, outcome definition, analytical approach, and interpretation of findings.

These revisions align the manuscript with the actual outcome analyzed and eliminate the inconsistency identified by the reviewer.

2. The sample derivation and missing-data explanation remain vulnerable. Excluding 14,040 observations is substantial, and the response should not overstate representativeness or claim minimal bias if many included/excluded variables differ significantly.

Response: Thank you for this important comment. We agree that the exclusion of 14,040 observations due to missing data is substantial and warrants careful consideration regarding potential selection bias and representativeness.

To address this concern, we compared the characteristics of included and excluded observations and found statistically significant differences for several variables. Accordingly, we have revised the manuscript to avoid overstating the representativeness of the final analytic sample or suggesting that the exclusions introduced only minimal bias. Instead, we explicitly acknowledge that these differences may have affected the composition of the study sample and could limit the generalizability of the findings.

In addition, we conducted further analyses to assess the potential impact of sample exclusion and have reported the corresponding results in the revised manuscript. While these analyses provide additional insight into the extent of potential bias, they cannot completely eliminate concerns arising from missing data and sample reduction.

To ensure transparency, we have expanded the description of sample derivation in the Methods section and added a dedicated discussion in the Limitations section. We now explicitly state that the complete-case analysis may be subject to selection bias because excluded observations differed from included observations on several characteristics. Therefore, the findings should be interpreted with appropriate caution and may not be fully representative of the entire target population.

3. The best-performing model is not consistently reported across the abstract, results, response letter, and tables. This should be corrected by specifying whether the “best” model is based on cross-validation, final test-set performance, AUROC, AUPRC, F1, or accuracy.

Response: Thank you for highlighting this inconsistency. To improve clarity and ensure consistent reporting throughout the manuscript, we have revised the abstract, results section, tables, and response letter to explicitly state that model performance was evaluated using six metrics: accuracy, precision, recall, F1-score, AUROC, and AUPRC. While all six metrics were considered in assessing model performance, AUROC was used as the primary reference metric for evaluating overall discriminatory ability.

Based on the combined evaluation of these performance measures, CART (using Lasso-selected features) and GBM (using Chi-square- and Boruta-selected features) demonstrated the strongest overall performance within their respective feature-selection approaches. In addition, the revised abstract now explicitly reports the best-performing model(s) together with their corresponding performance metrics (accuracy, precision, recall, F1-score, AUROC, and AUPRC) to provide a clear and transparent summary of model performance. We have ensured that the identification and reporting of the best-performing models are consistent across the abstract, results, tables, and supplementary materials.

Review Comments to the Author

Reviewer #1: The author has made improvements according to the input I provided very well, including improvements to the abstract, introduction, methods, results, discussion and conclusions. There are no more objections from me.

Response: Thank you for your positive assessment of our revised manuscript. We appreciate your careful review and valuable suggestions, which helped improve the quality, clarity, and overall presentation of the study. We are pleased that the revisions have satisfactorily addressed your concerns.

Reviewer #2: The authors appear to have made a reasonable effort to address the concerns raised by the reviewers. However, several of the revisions seem primarily aimed at providing justifications in response to reviewer comments rather than fully integrating the corresponding changes into the manuscript itself. Furthermore, the manuscript does not consistently adhere to the formatting requirements and guidelines of PLOS. The authors are encouraged to carefully review the journal's formatting policies and ensure full compliance prior to resubmission.

Response: Thank you for your thoughtful comments and for recognizing our efforts to address the concerns raised during the review process. We appreciate your observation that some revisions may have been more evident in the response letter than in the manuscript itself.

In response, we have carefully reviewed the entire manuscript and ensured that all substantive revisions described in the response letter have been fully incorporated into the manuscript text where appropriate. This includes revisions to the title, abstract, introduction, methods, results, discussion, limitations, and conclusions to ensure consistency, clarity, and transparency throughout the paper.

Additionally, we have conducted a thorough review of the manuscript to ensure compliance with PLOS ONE formatting requirements and submission guidelines. We have revised the manuscript accordingly, including formatting, section organization, figure and table presentation, references, and supplementary materials, to align with the journal’s requirements.

We appreciate this important recommendation and believe that these additional revisions have strengthened both the scientific content and the presentation of the manuscript.

Reviewer #3: All of the concerns appear to have been adequately addressed by the authors. I am confident that this review meets the standards required by PLOS One.

Response: Thank you for your encouraging comments and positive evaluation of our manuscript. We appreciate your careful review and are grateful for your assessment that the concerns raised during the review process have been adequately addressed. Your feedback has been invaluable in improving the quality of our work.

Attachment Submitted filename: Response to Reviewers.docx

# Peer review: Decision Letter 2

**DOI:** 10.1371/journal.pone.0356362.r005

**Authors:** Ammal Metwally

3 Aug 2026

Uncovering the Determinants of High-Risk Age at Childbirth in Bangladesh: A Machine Learning Analysis of the BDHS 2022 Data

PONE-D-25-49889R2

Dear Dr. Siam,

We’re pleased to inform you that your manuscript has been judged scientifically suitable for publication and will be formally accepted for publication once it meets all outstanding technical requirements.

Within one week, you’ll receive an e-mail detailing the required amendments. When these have been addressed, you’ll receive a formal acceptance letter and your manuscript will be scheduled for publication.

An invoice will be generated when your article is formally accepted. Please note, if your institution has a publishing partnership with PLOS and your article meets the relevant criteria, all or part of your publication costs will be covered. Please make sure your user information is up-to-date by logging into Editorial Manager at Editorial Manager® and clicking the ‘Update My Information' link at the top of the page. For questions related to billing, please contact billing support.

If your institution or institutions have a press office, please notify them about your upcoming paper to help maximize its impact. If they’ll be preparing press materials, please inform our press team as soon as possible -- no later than 48 hours after receiving the formal acceptance. Your manuscript will remain under strict press embargo until 2 pm Eastern Time on the date of publication. For more information, please contact onepress@plos.org.

Kind regards,

Ammal Mokhtar Metwally

Academic Editor

Additional Editor Comments (optional):

Reviewers' comments:

Reviewer's Responses to Questions

**Comments to the Author**

Reviewer #1: All comments have been addressed

Reviewer #2: All comments have been addressed

Reviewer #3: All comments have been addressed

**********

2. Is the manuscript technically sound, and do the data support the conclusions?

Reviewer #1: Yes

Reviewer #2: Yes

Reviewer #3: Yes

**********

3. Has the statistical analysis been performed appropriately and rigorously?

Reviewer #1: Yes

Reviewer #2: I Don't Know

Reviewer #3: Yes

**********

4. Have the authors made all data underlying the findings in their manuscript fully available?

The PLOS Data policy

Reviewer #1: Yes

Reviewer #2: Yes

Reviewer #3: Yes

**********

5. Is the manuscript presented in an intelligible fashion and written in standard English?

Reviewer #1: Yes

Reviewer #2: Yes

Reviewer #3: Yes

**********

Reviewer #1: We thank the authors for effectively revising the manuscript in accordance with our feedback.

Comments/Suggestion Journal Plos One

Uncovering the Determinants of Early Birth in Bangladesh: A Machine Learning Analysis of BDHS 2022 Dataset

Title:

• Ok

Abstract:

• ok

Introduction:

• ok

Methods

• Ok

Results

• Ok

Discussion:

• Ok

Conclusion

• Ok

Recommendation: Accept

Reviewer #2: (No Response)

Reviewer #3: I have no further comments to add. The authors have addressed all of the concerns that I believe were raised by reviewers.

**********

what does this mean?). If published, this will include your full peer review and any attached files.

If you choose “no”, your identity will remain anonymous but your review may still be made public.

**Do you want your identity to be public for this peer review?**  For information about this choice, including consent withdrawal, please see our Privacy Policy

Reviewer #1: No

Reviewer #2: **Yes:**  Deshani Chandima Kumari Herath

Reviewer #3: **Yes:**  Declan McKeown

**********

Attachment Submitted filename: Comments Reviewer-After Revision.docx

# Editor report: Acceptance letter

**DOI:** 10.1371/journal.pone.0356362.r006

**Authors:** Ammal Metwally

PONE-D-25-49889R2

PLOS One

Dear Dr. Siam,

I'm pleased to inform you that your manuscript has been deemed suitable for publication in PLOS One. Congratulations! Your manuscript is now being handed over to our production team.

At this stage, our production department will prepare your paper for publication. This includes ensuring the following:

* All references, tables, and figures are properly cited

* All relevant supporting information is included in the manuscript submission,

* There are no issues that prevent the paper from being properly typeset

You will receive further instructions from the production team, including instructions on how to review your proof when it is ready. Please keep in mind that we are working through a large volume of accepted articles, so please give us a few days to review your paper and let you know the next and final steps.

Lastly, if your institution or institutions have a press office, please let them know about your upcoming paper now to help maximize its impact. If they'll be preparing press materials, please inform our press team within the next 48 hours. Your manuscript will remain under strict press embargo until 2 pm Eastern Time on the date of publication. For more information, please contact onepress@plos.org.

You will receive an invoice from PLOS for your publication fee after your manuscript has reached the completed accept phase. If you receive an email requesting payment before acceptance or for any other service, this may be a phishing scheme. Learn how to identify phishing emails and protect your accounts at https://explore.plos.org/phishing.

If we can help with anything else, please email us at customercare@plos.org.

Thank you for submitting your work to PLOS ONE and supporting open access.

Kind regards,

PLOS ONE Editorial Office Staff

on behalf of

Professor Ammal Mokhtar Metwally

Academic Editor

PLOS One
